# Metagenomic Next-generation Sequencing in Patients With Infectious Meningoencephalitis: A Comprehensive Systematic Literature Review and Meta-analysis

**DOI:** 10.1093/ofid/ofaf274

**Published:** 2025-05-09

**Authors:** Pedro S Marra, Alexandre R Marra, Eileen Chen, Takaaki Kobayashi, Patrícia Deffune Celeghini, Maria Celidonio Gutfreund, Isabele Pardo, Gabriel O V Lopes, Mariana Kim Hsieh, Nicole A Boodhoo, Daniel Fu, Michael A Torres-Espinosa, Yimeng Li, Rodrigo Octávio Deliberato, Sulwan Mujahid A Algain, Jorge L Salinas, Michael B Edmond, Deyvid Emanuel Amgarten, Fernanda de Mello Malta, Nathalia Villa dos Santos, João Renato Rebello Pinho, Martineau Louine, Michael R Wilson

**Affiliations:** School of Medicine, University of California San Francisco, San Francisco, California, USA; Faculdade Israelita de Ciências da Saúde Albert Einstein, Hospital Israelita Albert Einstein, São Paulo, SP, Brazil; University of Iowa Health Care, Department of Internal Medicine, Iowa City, Iowa, USA; School of Medicine, University of California San Francisco, San Francisco, California, USA; University of Iowa Health Care, Department of Internal Medicine, Iowa City, Iowa, USA; Faculdade Israelita de Ciências da Saúde Albert Einstein, Hospital Israelita Albert Einstein, São Paulo, SP, Brazil; Faculdade Israelita de Ciências da Saúde Albert Einstein, Hospital Israelita Albert Einstein, São Paulo, SP, Brazil; Faculdade Israelita de Ciências da Saúde Albert Einstein, Hospital Israelita Albert Einstein, São Paulo, SP, Brazil; Faculdade Israelita de Ciências da Saúde Albert Einstein, Hospital Israelita Albert Einstein, São Paulo, SP, Brazil; Program of Hospital Epidemiology, University of Iowa Health Care, Iowa City, Iowa, USA; Department of Epidemiology, University of Iowa College of Public Health, Iowa City, Iowa, USA; Pritzker School of Medicine, University of Chicago, Chicago, Illinois, USA; School of Medicine, University of California San Francisco, San Francisco, California, USA; School of Medicine, University of California San Francisco, San Francisco, California, USA; Department of Biostatistics, Health Informatics and Data Science, University of Cincinnati College of Medicine, Cincinnati, Ohio, USA; Biomedical Informatics Division, Cincinnati Children's Hospital Medical Center, Cincinnati, Ohio, USA; Division of Infectious Diseases & Geographic Medicine, Stanford University, Stanford, California, USA; Division of Infectious Diseases & Geographic Medicine, Stanford University, Stanford, California, USA; Department of Medicine, West Virginia University School of Medicine, Morgantown, West Virginia, USA; Faculdade Israelita de Ciências da Saúde Albert Einstein, Hospital Israelita Albert Einstein, São Paulo, SP, Brazil; Faculdade Israelita de Ciências da Saúde Albert Einstein, Hospital Israelita Albert Einstein, São Paulo, SP, Brazil; Faculdade Israelita de Ciências da Saúde Albert Einstein, Hospital Israelita Albert Einstein, São Paulo, SP, Brazil; Faculdade Israelita de Ciências da Saúde Albert Einstein, Hospital Israelita Albert Einstein, São Paulo, SP, Brazil; LIM03/07, Hospital das Clínicas da Faculdade de Medicina da Universidade de São Paulo, São Paulo, SP, Brazil; Department of Neurology, Weill Institute of Neurosciences, University of California San Francisco, San Francisco, California, USA; Department of Neurology, Weill Institute of Neurosciences, University of California San Francisco, San Francisco, California, USA

**Keywords:** diagnostic methods, infectious meningoencephalitis, metagenomic next-generation sequencing, systematic literature review, *Mycobacterium tuberculosis*

## Abstract

**Background:**

We aimed to assess the accuracy, clinical efficacy, and limitations of metagenomic next-generation sequencing (mNGS) for diagnosing infectious meningoencephalitis.

**Methods:**

We performed a systematic literature review and meta-analysis of studies that evaluated the performance of mNGS to determine the cause of infectious meningoencephalitis. We explored PubMed, Cumulative Index to Nursing and Allied Health, Embase, Cochrane Central Register of Controlled Trials, ClinicalTrials.gov, and Web of Science up to 12 November 2024. To perform a meta-analysis, we calculated the pooled diagnostic odds ratio (DOR) for mNGS and for conventional microbiological tests (CMTs) compared to the clinical diagnosis.

**Results:**

Thirty-four studies met the inclusion criteria, with mNGS-positive rates ranging from 43.5% to 93.5% for infectious meningoencephalitis. The meta-analysis included 23 studies with 1660 patients. The pooled sensitivity was 0.70 (95% confidence interval [CI], .67–.72), and its specificity was 0.93 (95% CI, .92–.94). The DOR for mNGS was 26.7 (95% CI, 10.4–68.8), compared to 12.2 (95% CI, 3.2–47.0) for CMTs. For tuberculosis meningoencephalitis, mNGS demonstrated a pooled sensitivity of 0.67 (95% CI, .61–.72) and specificity of 0.97 (95% CI, .95–.99), with a DOR of 43.5 (95% CI, 7.4–256.6).

**Conclusions:**

Our review indicates that mNGS can be a valuable diagnostic tool for infectious meningoencephalitis, offering high sensitivity and specificity. mNGS's superior DOR compared to that of CMTs highlights its potential for more accurate diagnoses and targeted interventions. Further research is needed to optimize which patients and at what point in the diagnostic process mNGS should be used.

## Teaser

Our systematic review and meta-analysis show that metagenomic next-generation sequencing (mNGS) is superior to conventional microbiological test for diagnosing infectious meningoencephalitis, including tuberculosis meningoencephalitis, thus prompting further efforts to implement mNGS in clinical settings.

## Graphical Abstract

**Graphical Abstract:**
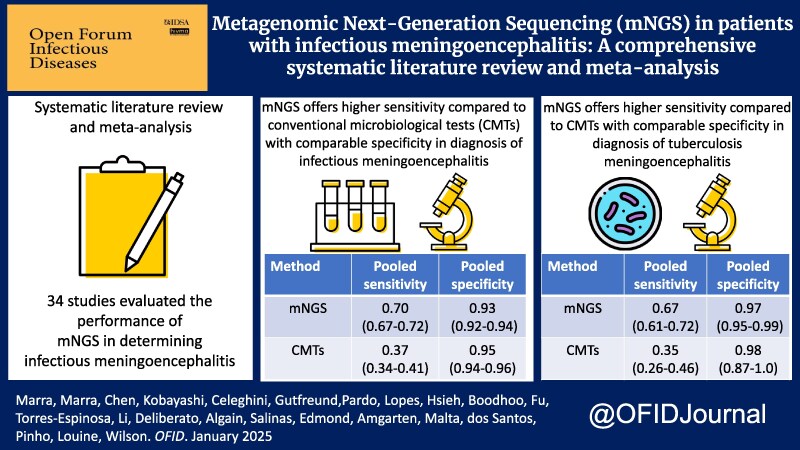

## BACKGROUND

Infectious meningoencephalitis is a severe and complex clinical condition [1]. The diagnosis is often challenging due to the wide range of potential pathogens, including bacteria, viruses, fungi, and parasites [1, 2]. Accurate and timely identification of the causative pathogen is important for effective treatment and improved patient outcomes. Traditional diagnostic methods, such as culture, serology, and polymerase chain reaction (PCR), can be limited by their inability to detect rare or difficult-to-culture organisms and by their time required to obtain results [3]. Metagenomic next-generation sequencing (mNGS) has recently emerged as a powerful diagnostic tool for infectious meningoencephalitis [3–8]. This technology allows for the simultaneous detection of a broad spectrum of pathogens in a single test, providing a comprehensive approach to pathogen identification. mNGS has the potential to transform the diagnostic process by detecting rare, novel, or unforeseen pathogens that may be overlooked by traditional methods. As mNGS technology is increasingly adopted in diagnostic laboratories worldwide, its utility in clinical practice continues to expand, offering significant advantages in terms of comprehensive pathogen detection and rapid turnaround times [3, 9].

Accordingly, the objective of this systematic literature review was to evaluate the performance of mNGS to determine the cause of infectious meningoencephalitis, particularly in comparison to conventional diagnostic methods. By synthesizing available evidence from published studies, we aim to assess the accuracy, clinical efficacy, and potential limitations of mNGS in this clinical context, ultimately providing insights into its role as a diagnostic tool for evaluating infectious meningoencephalitis.

## METHODS

### Systematic Literature Review and Inclusion and Exclusion Criteria

This review was conducted according to the Preferred Reporting Items for Systematic Reviews and Meta-Analysis statement [10] and the Meta-analysis of Observational Studies in Epidemiology guidelines [11]. This study was registered on Prospero (https://www.crd.york.ac.uk/PROSPERO/) on 8 June 2024 (registration number CRD42024554254). Institutional review board approval was not required. Clinical diagnosis of infectious meningoencephalitis includes taking a thorough patient history and performing physical examinations and diagnostic tests, such as imaging, blood tests, and tests of cerebrospinal fluid (CSF) [2]. For clarity, we will use the term “meningoencephalitis” throughout the paper, although we included studies that contained patients with meningitis, encephalitis, or both. mNGS is defined as a laboratory method that uses a random priming strategy to amplify and sequence all the genetic material in a sample to detect the full diversity of organisms present in a sample [3]. Conventional microbiological tests (CMTs) are a range of techniques including culture, serology, antigen tests, and pathogen-specific PCR assays. These tests collectively provide vital information for diagnosing infections, determining appropriate treatments, and tracking disease spread [3]. A positive result in CMTs was defined as at least 1 of the diagnostic methods mentioned previously being positive in a single sample.

This review included manuscripts published from the inception of each database up to 12 November 2024. There were no language restrictions. Inclusion criteria for studies in this systematic literature review were as follows: original research manuscripts; published in peer-reviewed, scientific journals; conducted in healthcare settings that evaluated the utilization of mNGS in patients with infectious meningoencephalitis with randomized clinical trial design; and observational study design. Commentaries, studies with overlapping individuals, studies with only pediatric patients, and studies in preprint were excluded.

### Search Strategy

**Figure 1.**
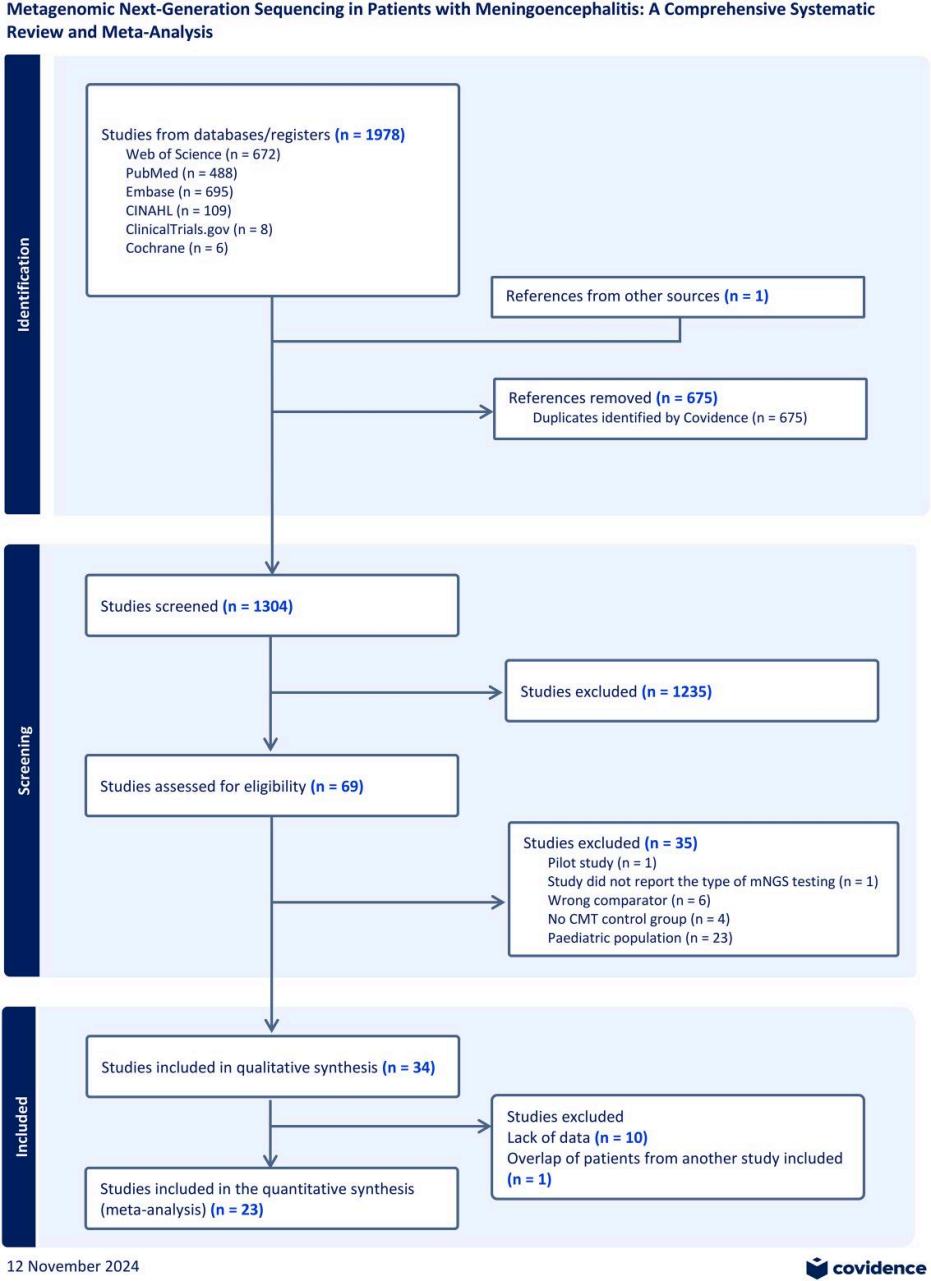
Literature search for articles on metagenomic next-generation sequencing (mNGS) in patients with meningoencephalitis.

We performed literature searches in PubMed, Cumulative Index to Nursing and Allied Health, Embase (Elsevier Platform), Cochrane Central Register of Controlled Trials, ClinicalTrials.gov, and Web of Science with an experienced health sciences librarian (E.C.) (Supplementary Appendix 1). We reviewed the reference lists of retrieved articles using Covidence [12] to identify studies that were not identified from the preliminary literature searches. To filter the 1640 articles obtained from the databases, titles and/or abstracts were assessed by 2 investigators (P.S.M. and A.R.M.) to exclude articles using the inclusion criteria. All disparities were resolved through consensus. After applying the exclusion criteria, we reviewed 69 studies, of which 34 met the inclusion criteria and were included in the systematic literature review (Figure 1). This study employs the PICO framework [13]. Focusing on patients classified as infectious meningoencephalitis (P), the study compares metagenomics (I) against CMTs (C). Our primary outcome of interest (O) was accuracy, clinical efficacy, and potential limitations of mNGS in this clinical context.

### Data Abstraction and Quality Assessment

Of 14 independent reviewers (P.S.M., A.R.M., T.K., P.D., M.C.G., I.P., G.O.V.L., M.K.H., N.A.B., M.T.E., Y.L., D.F., R.O.D., and S.M.A.A.), 2 independently abstracted data for each article using a standardized abstraction form (Supplementary Appendix 2). Reviewers resolved disagreements by consensus. All reviewers recorded data on study design, publication year and calendar time, population selection, and characteristics; mNGS methodology, sample type, and CMTs; the sensitivity and specificity of mNGS compared to clinical diagnosis, as well as the sensitivity and specificity of CMTs compared to clinical diagnosis; the sensitivity and the specificity of mNGS compared to CMTs; the type of the infectious meningoencephalitis and the most commonly detected pathogens; the advantages of mNGS over CMTs, and its clinical impact; and the limitations of mNGS. The risk of bias was assessed using the Downs and Black scale [14]. Reviewers answered all original questions except for question 27, which was modified to a yes or no answer. The highest possible score achievable on this scale was 28. Two authors performed the scale independently, and discrepancies were resolved by consensus.

### Patient Consent Statement

The present investigation is a systematic literature review of published deidentified data, so no patient informed consent was required.

### Statistical Analysis

To perform a meta-analysis on the extracted data (Supplementary Appendices 3 and 4), we calculated the pooled diagnostic odds ratio (DOR) for mNGS and for the CMTs in patients with infectious meningoencephalitis. The reference standard to calculate the DOR was clinical adjudication from the studies. Clinical adjudication (or clinical diagnosis) refers to the systematic review and interpretation of clinical data, including laboratory results, radiology findings, discharge summaries, and patient history, by a panel of expert clinicians, typically board-certified specialists in infectious diseases or relevant medical fields. This process aims to reach a consensus regarding the likelihood that a particular pathogen identified by diagnostic tests, such as mNGS or CMTs, is responsible for the patient's clinical condition, such as meningoencephalitis. Clinical adjudication served as the reference standard against which the diagnostic accuracy of mNGS and CMTs is compared, allowing for an assessment of their performance in identifying true positives, false positives, true negatives, and false negatives in diagnosing microbial infections [15].

We performed statistical analysis using R version 4.1.0 with mada package version 0.5.4 [16], and calculated the pooled sensitivity, specificity, and DOR. Analogous to the meta-analysis of the odds ratio method for the DOR, an estimator of random effects model following the approach of DerSimonian and Laird is provided by the mada package [16]. For our meta-analysis of estimates of diagnostic accuracy, we used a bivariate random effects model, which enabled simultaneous pooling of sensitivity and specificity with mixed-effect linear modeling while allowing for the tradeoff between them [17, 18]. Heterogeneity between studies was evaluated with *I*^2^ estimation and the Cochran Q statistic test. Publication bias was assessed using Egger's regression test with R version 4.1.0 with metafor package version 4.6-0 [19 ].

## RESULTS

### Characteristics of Studies Included in the Systematic Literature Review

Thirty-four studies met the inclusion criteria [20–53] and were included in the systematic literature review (Table 1). The vast majority of studies employed a retrospective cohort study design (22 studies) [20, 21, 25, 26, 31, 32, 34–36, 38, 39, 41, 42, 45–48, 50–53], although 12 were prospective cohort studies [22–24, 27–30, 37, 40, 43, 44, 49]. Most of the studies were conducted in China (27 studies) [21–26, 30–36, 39–42, 44–53] and in the United States (4 studies) [20, 37, 38, 43], including 1 in the United States with participants from Uganda [37], and 1 study was performed in each of the Netherlands [28], Switzerland [29], and Vietnam [27]. Studies were conducted between 2006 and 2023, varying in duration from 6 to 204 months [20–53]. In our qualitative analysis, 34 studies, including 3157 individuals with infectious meningoencephalitis, utilized various diagnostic approaches [20–53]. Of the 34 studies evaluated, 21 focused solely on DNA testing [21, 23–25, 30–35, 39, 41, 42, 44–48, 51–53], 12 evaluated both DNA and RNA testing [20, 22, 26, 27, 29, 36–38, 40, 43, 49, 50], and 1 focused solely on RNA testing [28]. The mNGS method involved primarily the sampling of CSF specimens, with only 1 study including CSF, blood, and other specimen types [29]. We only extracted data related to CSF samples in this study [29].

**Table 1.** Summary of Characteristics of Studies Included in the Systematic Literature Review

First Author, Year, Location, Study Design, Study Period in No. of Months and [dates]	Participants (n), and Characteristics	MNGS Methodology/ Sample Type, and Conventional Microbiological Tests	(Sensitivity, Specificity) of mNGS Compared to Clinical Diagnosis (Sensitivity, Specificity) of Conventional Microbiological Tests Compared to Clinical Diagnosis	(Sensitivity, Specificity) of mNGS Compared to Conventional Microbiological Tests	Type (s) of Meningoencephalitis and Most Common Detected Pathogens	Advantages of mNGS Over Other Diagnostic Methods and Clinical Impact	Limitations of mNGS	D&B Score (max = 28)
Benoit 2024 [20], USA Retrospective Cohort Study, 82** **mo (June 2016–April 2023)	1053 patients with suspected of infectious meningoencephalitis, 223 (19.2%) were diagnosed with infectious meningoencephalitis; clinical metadata analyzed of 1164 CSF samples. The mean age was 41.5 y, and 44% were female	DNA and RNA testing CSF Conventional tests included direct detection and serologic testing	mNGS: sensitivity = 63.1%, specificity = 99.6%; Conventional microbiological tests: Direct detection from CSF: sensitivity = 45.9%, specificity = 94.2%	mNGS sensitivity higher than other direct testing (*P* < .001)	Various infectious meningoencephalitis; most detected were DNA viruses (8.6%), RNA viruses (4.4%), bacteria (2.9%), fungi (2.2%), and parasite (0.5%)	Higher sensitivity and detection rate of pathogens; supports routine diagnostic workup for CNS infections	mNGS sensitivity lower than direct detection in some instances; dependent on sample quality	19
Chen 2022 [21], China Retrospective Cohort Study, 25** **m (January 2019–February 2021)	216 participants with tuberculosis meningitis, being 88 as definite, 5 as probable, and 24 as possible tuberculosis cases. The median age was 40 (IQR 25–43) years. Gender was not reported	DNA testing CSF CSF mycobacterial culture; modified Ziehl-Neelsen stain; PCR (Xpert MTB/rifampicin)	mNGS: sensitivity = 84%, specificity = 100%; Conventional microbiological tests: Culture: sensitivity = 25%, specificity = 100%; Ziehl-Neelsen stain = sensitivity = 76.1%, specificity = 100%; PCR = sensitivity = 73.9%, specificity = 100%	NR	Tuberculosis *Mycobacterium tuberculosis*	mNGS provides a substantial improvement in accurate diagnosis of tuberculosis meningitis	NR	19
Chen 2024 [22], China Prospective Cohort Study, 24 m (January 2021–December 2022)	Of 152 patients with suspected meningoencephalitis, 89 were diagnosed with infectious meningoencephalitis. The median age was 49 (IQR 18–83) years, and 45% out of 89 were female	DNA and RNA testing CSF Microbiologic methods include Gram staining or culture for bacterial analytes; nucleic acid amplification tests or antibody tests in CSF for viral analytes; mycobacterial staining, culture and Xpert MTB/RIF for mycobacterial analytes; and cryptococcal antigen or fungal culture for *Cryptococcus neoformans/gattii*	mNGS: sensitivity = 43.8%, specificity = 52.4%; targeted mNGS: sensitivity = 64%, specificity = 66.7%; Conventional microbiological tests: NR	NR	Bacterial, viral, fungal, and tuberculosis The most commonly detected pathogens were viruses (77.5%), with HSV-1 being the most prevalent (36%)	Targeted mNGS seems to have more potential than mNGS for detecting common virus pathogens, with detection rates of 65% and 46%, respectively	mNGS data requires careful analysis to distinguish genuine pathogens from contaminants to avoid spurious disease associations	17
Deng 2023 [23], China Prospective Cohort Study, 12 m (January 2021–January 2022)	Among 1131 individuals living with HIV/AIDS who were hospitalized, 48 had meningoencephalitis and were investigated using mNGS versus conventional microbiological tests. The mean age was 40 (SD ± 13.4) y, and 8.3% of 48 were female	DNA testing CSF CSF conventional tests include smears and cultures for bacteria, fungi, and *Mycobacterium*, along with specific tests like cryptococcal antigen, Chinese ink staining, and GeneXpert MTB/RIF for common infections	mNGS: sensitivity = 63.9%, specificity = 66.7%; Conventional microbiological tests: sensitivity = 36.0%, specificity = 23.8%	NR	Bacterial, viral, fungal, tuberculosis, and parasitic Top 3 pathogens: EBV, CMV, and *Cryptococcus neoformans*	mNGS was significantly superior to clinical conventional method in bacteria, viruses, and atypical pathogens detection. The combination of mNGS and conventional microbiological tests can improve the sensitivity and sensitivity of meningoencephalitis diagnosis in individuals living with HIV/AIDS	mNGS was less effective in detecting fungal infections and struggled with identifying mycobacterium, including tuberculosis. Additionally, mNGS carries a high economic burden	21
Fu 2024 [24], China Prospective Cohort Study, 6 m (July 2021–December 2021)	Of 50 patients with suspected meningoencephalitis, 31 were diagnosed with infectious meningoencephalitis. The median age of all participants was 57 y. Females comprised 50% of the participants	DNA testing CSF Microbial culture	mNGS: sensitivity = 87.1%, specificity = 43.8%; Conventional microbiological tests: NR	NR	Bacterial The most commonly detected pathogens were *Burkholderia cepacia* (25%), followed by *S aureus* (20.5%), *Klebsiella pneumoniae* (10.2%), and *E coli* (8.0%)	NR	NR	18
Gan 2022 [25], China Retrospective Cohort Study, 18 m (July 2018–December 2019)	Of 197 HIV-negative patients with suspected central nervous system infections, 46 were diagnosed with cryptococcal meningitis. The mean age was 45 (SD ± 32.6) y, and 32.6% of 46 were female	DNA testing CSF India ink staining, fungal culture, or cryptococcal antigen tests	mNGS: sensitivity = 93.5%, specificity = 96.0%; Conventional microbiological tests: India ink: sensitivity = 25%, specificity = 100%	mNGS versus culture: sensitivity = 100%, specificity = 90.5%	Fungal *Cryptococcus neoformans, Cryptococcus gattii*	The sensitivity of mNGS for the diagnosis of cryptococcal infections was slightly lower than that of cryptococcal antigen test but higher than those of India ink and culture, and its specificity was slightly lower than that of the traditional methods, with the concordance rate between that of cryptococcal antigen test and culture	mNGS tests are currently very expensive and slower compared to India ink staining or cryptococcal antigen tests. Additionally, antifungal drug resistance analysis is not available. Therefore, mNGS cannot replace traditional diagnostic tests for CM and is not recommended for routine use	18
Gu 2022 [26], China Retrospective Cohort Study, 24 m (January 2019**—**December 2020)	Of 66 patients with suspected meningoencephalitis, 41 were diagnosed with infectious meningoencephalitis. The median age was 49 (IQR 18–83) years, and 45% of 89 were female	DNA and RNA testing CSF NR	mNGS: sensitivity = 65.8%, specificity = 71.4%; Conventional microbiological tests: NR	mNGS versus CMT (not reported which CMT) for bacterial meningitis [n = 13]: sensitivity = 40%, specificity = 25% mNGS versus CMT (not reported which CMT) for viral meningoencephalitis [n = 18]: sensitivity = 100%, specificity = 62.5%	bacterial, viral, fungal, and tuberculosis The cases included 18 viral infections (43.9%), 13 bacterial infections (31.7%), 3 *Mycobacterium tuberculosis* infections (7.3%), 5 fungal infections (12.2%), and 2 *Rickettsia* infections (4.9%)	mNGS technology excels in detecting rare or unknown pathogens that targeted detection methods might miss. It can diagnose conditions like *Pseudorabies* virus encephalitis in CSF and severe viral encephalitis in humans. Additionally, it identified *Rickettsia felis* infections in 2 patients, both of whom improved after appropriate treatment	In some cases of viral encephalitis, meningitis, and noninfectious diseases, multiple bacteria and fungi were detected, likely due to sample contamination. EBV and human polyomavirus type 5 were found in a few cases but were considered false positives despite meeting detection standards.	20
Hong 2020 [27], Vietnam Prospective Cohort Study, 21** **mo (January 2015–September 2016)	66 participants with viral meningoencephalitis. The median age was 35 (IQR 15–84) years, and 41% out of 66 were female	DNA and RNA testing CSF CSF samples were tested for bacterial, fungal, or *Mycobacterium tuberculosis* infections. HSV PCR was used for suspected meningoencephalitis, and additional tests for VZV, dengue, Japanese encephalitis, or MuV were done if necessary.	NR	mNGS versus PCR: sensitivity = 73.7%, specificity = 66.0%	Viral The most commonly detected viruses were Enterovirus, HSV, and mumps virus	NR	There are no robust criteria to define a true mNGS positive without confirmatory testing. Proposed criteria include at least 3 reads mapped to different genomic regions of a virus or absence of viral reads in negative controls. Cross-talk contamination, a known issue in mNGS, can be significantly reduced with the newly developed dual barcoding strategy	20
Kinsella 2022 [28], Netherlands Prospective Cohort Study, 204 m (2006–2022)	2705 participants with bacterial meningitis were included in the MeninGene study, being 89 patient samples from this cohort for VIDISCA analysis The median age was 58 (IQR 34–67) years, and 44% out of 89 were female	RNA testing CSF CSF culture	mNGS (all pathogens, read identify cutoff ≥97%, and minimum pathogen read = 1): sensitivity = 89.0%, specificity = 57.0%; Conventional microbiological tests: NR	NR	Bacterial The most commonly detected bacteria were *Streptococcus pneumoniae, Listeria monocytogenes, Neisseria meningitidis*	The study detected both nonpathogenic and pathogenic viruses in CSF samples. EV-D68 and HIV-1 were found in patients with bacterial meningitis, but the clinical significance of these findings is uncertain. EV-D68 was associated with gastrointestinal symptoms in a patient co-infected with L. monocytogenes, though the exact cause of the symptoms is unclear	A high number of false-positive detections of *S pneumoniae*, a problem also seen with multiplex PCR panels, likely due to high carriage rates in the population	19
Kufner 2019 [29], Switzerland Prospective Cohort Study, 26 m (May 2017–June 2019)	105 participants with suspected infectious diseases but only 67 with clinical data, being 17 patients with meningoencephalitis The median age was 53 (range 17–88) years, and 36% out of 67 were female	DNA and RNA testing CSF, blood, and other samples Different diagnostic panels (ePlex Respiratory Pathogen Panel, GeneXpert Flu/RSV, Fast-Track Diagnostics Respiratory Pathogens 21, RIDAGENE Gastrointestinal Panel) and individual tests were assessed. The average number of “diagnostic requests” per patient was calculated, encompassing various virus detection methods including specific PCR, immunofluorescence, serology, culture, and intrathecal antibody synthesis testing	NR	mNGS versus CMTs: sensitivity = 88.0%, specificity = 93.0%	Viral Not reported the prevalence of viral infections	Untargeted approach, rapid and efficient virus diagnostics, confirming the utility of mNGS in complementing current routine tests	Due to the lower sensitivity of mNGS compared to specific PCR, specific tests will still be needed initially, especially for pathogens requiring rapid diagnosis and treatment. Close interdisciplinary collaboration with clinicians is essential to contextualize the findings, as multiple viruses are often detected	19
Lin 2021 [30], China Prospective Cohort Study, 6 mo (February 2018**—**August 2018)	50 participants of clinically suspected tuberculosis meningitis NR	DNA testing CSF Cerebrospinal fluid tests for *M tuberculosis* include culture, smear, MTB nucleic acid PCR test, and the Xpert MTB/RIF test	mNGS: sensitivity = 58.8%, specificity = 100%; Conventional microbiological tests (culture and PCR): sensitivity = 29.4%, specificity = 100%	mNGS versus CMTs: sensitivity = 80.0%, specificity = 70.0%	tuberculosis	mNGS combined with *M tuberculosis* culture could increase the detection rate	High cost; difficulties in specimen preparation process; and inability of mNGS to directly detect the resistance of *M tuberculosis* strains	19
Lin 2023 [31] [AJTR], China Retrospective Cohort Study, 57 m (March 2014**—**December 2018)	Of 211 patients with suspected meningoencephalitis, 201 were diagnosed with infectious meningoencephalitis. The median age was 44 (IQR 29–57) years for the mNGS group and was 46 (IQR 31–57.5) years for the conventional microbiological test group. Females made up 22.7% in mNGS group and 39.8% of the conventional microbiological test group	DNA testing CSF CSF smear, and the traditional culture of bacteria, fungi, and tuberculosis	mNGS: sensitivity = 91.7%, specificity = 88.0%; Conventional microbiological tests: NR	NR	Bacterial, viral, fungal, tuberculosis, and parasitic The most commonly detected pathogens were: HSV, *M tuberculosis*, *Klebsiella pneumoniae,* and *Candida parapsilosis*	mNGS proved superior to traditional cultures in detecting meningoencephalitis, leading to shorter hospital stays and improved patient outcomes, particularly in severe cases and bacterial infections. The use of mNGS also reduced daily drug intensity in patients, which helps prevent antibiotic resistance and ensures better management of meningoencephalitis	NR	21
Lin 2023 [32] [DMID], China Retrospective Cohort Study, 12 mo (April 2020**—**April 2021)	605 individuals were HIV- negative, 280 had suspected tuberculosis meningitis, being 10 as definite, 18 as probable, and 170 as possible tuberculosis cases. The mean age was 48.4 y. Female was reported in 30% of definite *M. tuberculosis* cases	DNA Testing CSF AFB staining, culture, *M tuberculosis* PCR, Xpert MTB/RIF	mNGS: sensitivity = 70.0%, specificity = 95.2%; Conventional microbiological tests: AFB: sensitivity = 10.0%, specificity = 100%	NR	Bacterial, viral, fungal, and tuberculosis Top 3 pathogens: *M tuberculosis*, *Cryptococcus neoformans,* and HSV	It has the ability to detect all pathogens in a sample simultaneously, facilitating simultaneous diagnosis of multiple infectious diseases	NR	20
Lin 2024 [33], China Retrospective Cohort Study, 27 mo (January 2021**—**March 2023	Of 40 participants, 34 were diagnosed as tuberculosis meningitis, being 15 as definite, and 19 as clinical diagnosis based on symptoms, CSF, imaging, pathogens, and treatment. The mean or median age was not reported. Female was reported in 40% of definite *M tuberculosis* cases	DNA testing CSF Acid-fast bacilli (AFB) staining, culture, *M tuberculosis* PCR, Xpert MTB/RIF	mNGS: sensitivity = 78.6%, specificity = 100%; Conventional microbiological tests: NR	NR	Tuberculosis	Higher sensitivity	Most patients had the mNGS tested only once. Testing more times could increase sensitivity. The CSF of patients had not undergone all the *M tuberculosis* tests simultaneously	17
Liu 2023 [34], China Retrospective Cohort Study, 12 mo (January 2020**—**December 2020)	Of 94 participants, 43 were diagnosed as infectious meningoencephalitis. The median age of all participants was 44 (range 0.25–82) years. Females comprised 39% of the participants	DNA testing CSF CSF samples underwent routine tests including bacterial and fungal smears, acid-fast staining, cultures, and viral testing. PCR was used for suspected tuberculous encephalitis or meningitis, and cryptococcal meningitis was assessed with cryptococcal antigen staining	NR	mNGS versus CMTs: sensitivity = 82.4%, specificity = 62.3%	bacterial, viral, fungal, and tuberculosis Gram-negative bacteria (n = 16), Gram-positive bacteria (n = 11), viruses (n = 10), fungi (n = 4), and one special pathogen (*M avium*) were detected by mNGS alone	The results show that mNGS outperforms traditional methods in detecting both the species and quantity of pathogens, particularly fungi, mycobacteria, and viruses. It is also more effective at diagnosing mixed infections and infections with specific pathogens. Additionally, mNGS offers a faster turnaround time compared to conventional methods	NR	18
Lu 2024 [35], China Retrospective Cohort Study, 42 mo (January 2018**—**July 2021)	195 participants with suspected viral CNS infection, being 130 with meningoencephalitis. The mean age of all participants was 41.5 (SD ± 16.9) y. Females comprised 34% of the participants	DNA testing CSF PCR or antibody detection	mNGS: sensitivity = 91.7%, specificity = 90.1%; Conventional microbiological tests: NR	NR	Viral VZV (28.0%) was the most common virus detected by mNGS in 175 patients with CNS viral infections, followed by HSV-1 (18.3%) and EBV (7.4%).	mNGS using cell-free DNA is more effective than using wcDNA due to reduced human DNA interference. Despite mNGS's ability to identify DNA viruses, particularly herpesviruses, qPCR assays were less successful, with only 22.9% positivity. This indicates that mNGS is more sensitive than qPCR for CSF infections. Identifying the cause of CNS disease requires comprehensive analysis, including epidemiological history, clinical presentation, imaging, CSF, and serological findings, alongside NGS reports	RNA virus detection was not conducted, potentially causing false negatives. RNA viruses like enteroviruses are common causes of encephalitis/meningitis False negatives may occur due to improper specimen collection, transportation, and preservation	20
Pan 2024 [36], China Retrospective Cohort Study, 39 m (January 2019**—**March 2022)	54 participants with suspected CNS infection, being 20 with meningoencephalitis Mean age was 30.2 (SD ± 14.25) years in viral meningoencephalitis cases Female was reported in 40% of all types of meningoencephalitis cases	DNA and RNA testing CSF Conventional pathogen tests performed but not specified	mNGS: sensitivity = 80.0%, specificity = 97.1%; Conventional microbiological tests: NR	NR	Bacterial, viral, fungal, and tuberculosis The most cases detected by mNGS were viral infections (8/11, 73%)	mNGS is a highly sensitive, comprehensive, and timely detection method that helps clinicians quickly identify pathogens, initiate targeted treatments early, and reduce antibiotic misuse	The clinical application of mNGS faces issues such as lack of standardization in the experimental process, absence of quality control standards for bioinformatics analysis, and the subjective interpretation of results	22
Ramachandran 2022 [37], USA [participants from Uganda] Prospective Cohort Study, 24 mo (March 2018–March 2020)	Of 368 participants in the study with suspected infectious meningoencephalitis, nearly half were diagnosed with cryptococcal meningitis (not-tuberculosis meningitis group). The median age was 35 (IQR 29–41) y with 42.7% female	DNA and RNA testing CSF CSF samples underwent routine tests including bacterial and fungal smears, acid-fast staining, cultures, and viral testing. PCR was used for suspected tuberculous encephalitis or meningitis, and cryptococcal meningitis was assessed with cryptococcal antigen staining	Tuberculosis was detected by mNGS in 74.4% (29/39) of confirmed tuberculosis meningitis cases; Conventional microbiological tests: NR	NR	bacterial, viral, fungal, tuberculosis, and parasitic Besides cryptococcal and tuberculosis cases, 19 neuroinvasive viral infections (other than HIV) were detected, along with 9 cases of bacterial meningitis (including 4 cases of *S pneumoniae*), and 15 cases of *T gondii*	mNGs identified several underdiagnosed but treatable neurologic infections. Machine learning classifiers were used to help improve diagnostic accuracy of tuberculosis meningitis	mNGS not readily available in Uganda and samples were sent to the USA for testing	19
Steinberg 2023 [38], USA Retrospective Cohort Study, 68 mo (August 2014**—**March 2020)	140 participants with HIV, being 40 with meningoencephalitis (toxoplasmosis [27 cases] and cryptococcal meningitis [13 cases] The median age was 34 y (range 28–40) for toxoplasmosis cases, with 23% being female. For cryptococcal meningitis, the median age was 36 y (range 32–41), with 8% being female	DNA and RNA testing CSF *Toxoplasma gondii* qPCR, and cryptococcal antigen	NR	mNGS versus qPCR (*Toxoplasma gondii*): sensitivity = 71.4%, specificity = 91.7%; mNGS versus cryptococcal antigen (serum or CSF): sensitivity = 78.0%, specificity = 100%	fungal and parasitic *Toxoplasma gondii* and *Cryptococcus neoformans*	NR	NR	20
Sun 2021 [39], China Retrospective Cohort Study, 36 mo (January 2017**—**December 2019)	Clinical and microbiological data were analyzed for 208 patients with tuberculosis smear-negative extrapulmonary specimens, of whom 180 were diagnosed with extrapulmonary tuberculosis, including 45 with tuberculosis meningitis. The mean age was 29.6 (SD ± 18.1) y, and 42.7% of 180 were female	DNA testing CSF AFB staining, culture, *M tuberculosis* PCR, Xpert MTB/RIF	mNGS: sensitivity = 84.4%, specificity = NR; Conventional microbiological tests: NR	NR	Tuberculosis (45 cases of tuberculosis meningitis) Out of the 28 non-extrapulmonary tuberculosis cases, mNGS identified 2 cases of fungal meningitis, and one case of amoeba meningitis	The sensitivity of mNGS varied markedly across the different specimen types, being the highest for tuberculosis meningitis (84.4%). It demonstrated consistent diagnostic capability regardless of prior treatment. Additionally, mNGS has the potential to identify other infections and co-infections simultaneously, with a reporting time of just 48 h	It requires a large amount of information which also requires professional bioinformatics personnel to analyze data, leading to high cost	17
Tian 2024 [40], China Prospective Cohort Study, 15 mo (December 2021**—**December 2023)	139 participants with suspected CNS infection after neurosurgical procedure in patients admitted in ICU, being 66 with meningoencephalitis Mean age was 40.8 (SD ± 21.3) y and 38% were female	DNA and RNA testing CSF Pathogen culture	mNGS: sensitivity = 63.6%, specificity = 98.6%; Conventional microbiological tests: sensitivity = 30.3%, specificity = 100%	NR	Bacterial, viral Most common pathogens: *Acinetobacter baumannii*, *Acinetobacter necromimesis*, *Klebsiella pneumoniae* Human herpervirus 6B (1 case)	mNGS and pathogenic cultures were conducted within 24 h of suspected CNS infection The mNGS also detected bacterial spectrum and antimicrobial resistance genes Metagenomics has the potential to assist in the diagnosis of patients with CNS infections who have a negative culture	High cost of mNGS testing	18
Wang 2019 [41], China Retrospective Cohort Study, 34 mo (December 2015**—**October 2018)	23 participants with tuberculosis meningitis. Mean or median age not reported, and 30% were female	DNA testing CSF Culture of *Mycobacterium tuberculosis*, PCR, and AFB stain	NR	mNGS versus PCR: sensitivity = 66.7%, specificity = 100%	tuberculosis	In patients with definite TBM, the sensitivity of mNGS, AFB, PCR, and culture for detecting MTB in the initial CSF samples was 66.67%, 33.33%, 25%, and 8.33%, respectively, with all methods having 100% specificity. Combining mNGS with conventional methods increased the detection rate to 95.65%	mNGS was performed only once per patient due to its high cost, limiting its widespread use and repeated detection. Since the timing of CSF collection may not align with the peak pathogen occurrence, repeating mNGS tests could enhance sensitivity.	16
Wang 2022 [42], China Retrospective Cohort Study, 48 m (2018–2021)	41 participants with suspected CNS infection, being 31 with meningoencephalitis Median age was 47.7 (4–75) y and 26% were female	DNA testing CSF Bacterial, fungal, and tuberculosis cultures, autoimmune antibody tests, serologic tests, and CSF smears were performed as needed. Cell biopsies and nucleic acid amplification tests (including traditional PCR and Xpert MTB) were conducted based on clinical assessment	mNGS: sensitivity = 54.8%, specificity = 60.0%; Conventional microbiological tests: sensitivity = 32.3%, specificity = 90.0%	mNGS versus CMTs: sensitivity = 90.9%, specificity = 63.3%	Bacterial, viral, fungal, and tuberculosis Most cases were tuberculosis. Other species were not reported	NR	Contamination during sampling and laboratory operations can lead to false-positive results by introducing unrelated microorganisms. Additionally, the type and cellularity of the pathogen can impact the detection rate	18
Wilson 2019 [43], USA Prospective Cohort Study, 13 mo (June 2016**—**July 2017)	Of 204 hospitalized participants in the study with suspected meningoencephalitis, 28% were diagnosed with infectious meningoencephalitis. The mean age of all patients was 39.6 y, and 44% were female	DNA and RNA testing CSF Discrepant results were confirmed using either a validated clinical assay or PCR testing in a research laboratory	mNGS: sensitivity = 80.0%, specificity = 98.2%; Conventional microbiological tests: sensitivity = 67.5%, specificity = 99.4%	NR	Bacterial, viral, and fungal 13 cases diagnosed by mNGS only (eg, *N farcinica, Candida tropicalis*, hepatitis E virus)	mNGS offers an unbiased approach to detect multiple potential infectious agents in a single assay without the need for prior selection of targeted pathogens. This makes it particularly useful for diagnosing infections in CSF samples, where sample volume is often limited. Even when mNGS results align with conventional tests, it provides confirmation of the diagnosis and can detect or rule out coinfections, which is especially valuable in immunocompromised patients	NR	21
Xing 2020 [44], China Prospective Cohort Study, 30 mo (November 2016**—**May 2019)	213 participants with suspected CNS infection, being 180 with meningoencephalitis The median ages for viral, tuberculosis, bacterial, and fungal meningitis were 30 (14–64), 41 (14–76), 44 (14–76), and 54.5 (15–80) y, respectively. The percentages of females were 39%, 43%, 33%, and 28%, respectively	DNA testing CSF Conventional microbiological test for viral, bacterial, tuberculosis, and fungal meningitis	NR	mNGS versus CMTs: sensitivity = 85.7%, specificity = 96.4%	Bacterial, viral, fungal, and tuberculosis	mNGS of CSF effectively identifies pathogens causing infectious CNS diseases and should be used alongside conventional microbiological testing	RNA-Seq data were not analyzed alongside DNA sequencing, which could have offered valuable complementary insights.	16
Yan 2020 [45], China Retrospective Cohort Study, 24 mo (January 2017**—**December 2018)	Of 51 participants, 45 were diagnosed as tuberculosis meningitis, being 38 as definite, 5 as probable, and 2 as possible. The median age was 34 (IQR 19–80) y. Female was reported in 20% of *M tuberculosis* cases	DNA testing CSF Culture of *Mycobacterium tuberculosis*, AFB stain, PCR, and Xpert MTB/RIF	mNGS (final diagnosed cases of tuberculosis meningitis): sensitivity = 84.4%, specificity = 100%; Conventional microbiological tests (final diagnosed cases of tuberculosis meningitis): sensitivity = 0, 22%, 24%, 40%, for AFB, culture, PCR, Xpert MTB/RIF, respectively, specificity = 100% for AFB, culture, PCR, Xpert MTB/RIF	NR	Tuberculosis, fungal mNGS in CSF samples yielded etiological positive results in 41 cases (80.4%, 41/51), including *M. tuberculosis* in 38 cases and fungi in 3 cases (2 *Candida* and 1 *Aspergillus niger*)	mNGS demonstrated higher sensitivity and specificity in diagnosing definite, probable, and possible cases of TBM compared to standard diagnostic tests such as AFB and Xpert	mNGS testing is not covered by public medical insurance and is not considered routine or repeatable, so each patient was tested only once. The study had a smaller sample size due to the financial constraints associated with using mNGS	17
Yu 2021 [46], China Retrospective Cohort Study, 12 mo (May 2019**—**April 2020)	Of 37 participants, 23 were diagnosed with tuberculosis meningitis, including 4 with definite, and 19 with probable cases. The median age was 66 (IQR 36–75.8) years for definite cases, and 53 (IQR 32–71) years for probable cases. Female accounted for 35% of *M tuberculosis* cases	DNA testing CSF AFB staining, culture, *M tuberculosis* PCR, Xpert MTB/RIF	mNGS: sensitivity = 43.5%, specificity = 100%; Conventional microbiological tests: sensitivity = 34.8%, specificity = 100%	mNGS versus CMTs: sensitivity = 75.0%, specificity = 86.0%	Tuberculosis	In patients with unexplained meningitis, mNGS can be used as initial test to quickly identify the cause of the disease because it is not limited to detecting only *M tuberculosis*	NR	17
Yu 2022 [47], China Retrospective Cohort Study, 36 mo (June 2018**—**June 2021)	CSF samples from 390 patients clinically diagnosed with infectious meningoencephalitis were used for the mNGS of cell-free DNA (n = 394) and whole-cell DNA (n = 150). The mean age of all patients was 43.6 (SD ± 1.7) years, and 34% were female.	DNA testing CSF CSF samples underwent routine tests including bacterial and fungal smears, acid-fast staining, cultures, and viral testing. PCR was used for suspected tuberculous encephalitis or meningitis	mNGS: sensitivity = 60.2%, specificity = NR; Conventional microbiological tests: sensitivity = 20.9% %, specificity = NR	NR	Bacterial, viral, fungal, tuberculosis, and parasitic *Streptococcus pneumoniae* infections were more likely to lead to severe cases of meningitis and encephalitis, while Human alpha herpesvirus 3 typically caused less severe forms of the disease	Both cell-free DNA mNGS (60%) and whole-cell DNA mNGS (32%) showed higher sensitivity compared to conventional methods (20.9%). mNGS was more effective at identifying a broader range of causative pathogens, while conventional methods were more limited	NR	18
Yuan 2024 [48], China Retrospective Cohort Study, 60 mo (Feb 2018**—**Feb 2023)	Of 111 participants, 64 had infectious meningoencephalitis. The mean age of these patients was 47.9 (range 18–76) years, and 33% were female	DNA testing CSF CSF samples underwent routine tests including bacterial and fungal smears, acid-fast staining, cultures, and viral testing. PCR was used for suspected tuberculous encephalitis or meningitis	mNGS: sensitivity = 68.7%, specificity = 82.9%; Conventional microbiological tests: sensitivity = 26.5%, specificity = 95.7%	NR	Bacterial, viral, fungal, and tuberculosis Top 3 pathogens: *M tuberculosis,* EBV*, Klebsiella pneumoniae*	Higher detection rate of mNGS in infectious meningoencephalitis with CSF white blood cell count >300 × 10^6^L; better sensitivity compared to conventional microbiological tests	Conventional tests insufficient for all pathogens; potential issues with false positives/negatives	19
Zhang 2020 [49], China Prospective Cohort Study, 12** **mo (February 2017**—**February 2018)	Of 230 participants, 159 had infectious meningoencephalitis. The mean age of these patients was 44.4 (range 13–73) years, and 39% were female	DNA and RNA testing CSF CSF was sent for smear, culture, PCR, special antibody tests	mNGS: sensitivity = 69.3%, specificity = 98.6%; Conventional microbiological tests: sensitivity = NR, specificity = 100%	NR	Bacterial, viral, fungal, tuberculosis, and parasitic Top 3 pathogens: Klebsiella pneumoniae, *M tuberculosis*, and Acinetobacter baumannii. The most detected fungal infection was cryptococcal meningitis, and the most detected viral was human herpes virus 3	mNGS outperformed conventional methods in detecting bacterial meningoencephalitis, though it showed no advantage for viral, fungal, or parasitic infections. For patients who have received empirical antimicrobial treatment, mNGS offers a clear advantage over conventional methods for CSF testing. Conventional tests rely on detecting living pathogens, which can be challenging after treatment begins	mNGS improves diagnostic efficacy if used within four days of starting treatment, but its effectiveness significantly declines after this period	20
Zhang 2024 [50], China Retrospective Cohort Study, 55 mo (March 2018**—**September 2022)	Of 90 participants with suspected infectious meningoencephalitis, 67 had infectious meningoencephalitis. The mean age of all participants was 45.7 (range 18–78) years, and 32% were female	DNA and RNA testing CSF CSF conventional tests include smears and cultures for bacteria, fungi, and *Mycobacterium*, along with specific tests like cryptococcal antigen, Chinese ink staining, and GeneXpert MTB/RIF for common infections	mNGS: sensitivity = 61.5%, specificity = 88.0%; Conventional microbiological tests: NR	NR	Bacterial, viral, fungal, and tuberculosis The cases included 71.6% viral infections, 12.2% bacterial infections, 7.5% *Mycobacterium tuberculosis*, 3.0% fungal infections, and 1.5% rickettsia infection	mNGS can rapidly detect rare pathogens, such as *Orientia tsutsugamushi*, which are rarely associated with infectious meningoencephalitis. It outperforms conventional methods, especially in patients pre-treated with antibiotics, by identifying a broader range of pathogens more quickly	Strict adherence to sterile procedures is essential to prevent sample misidentification or contamination, which can lead to false-positive results	20
Zhao 2020 [51], China Retrospective Cohort Study, 18 mo (March 2018**—**September 2019)	Of 104 patients with suspected meningoencephalitis, 49 were diagnosed with infectious meningoencephalitis. The mean or the median age of all participants was not reported, and 41% were female	DNA testing CSF NR	mNGS: sensitivity = 59.0%, specificity = NR; Conventional microbiological tests: NR	NR	Bacterial, viral, fungal, and parasitic 94% of infectious meningoencephalitis cases were caused by bacteria and viruses	mNGS effectively diagnosed cases of infectious meningoencephalitis, offering strong support for physician bedside diagnoses and decision-making compared to traditional methods	Unlike traditional methods like serology and PCR, mNGS often detects large amounts of background microbial genetic material, making it crucial to identify the relevant pathogen. Sample collection and testing can also introduce contamination	14
Zhu 2022 [52], China Retrospective Cohort Study, 48 mo (March 2018**—**September 2022)	Among 80 individuals living with HIV/AIDS who were hospitalized, 73 had meningoencephalitis and were investigated using mNGS. The median age was 37 (IQR 31–49) years, and 10% out of 80 were female	DNA testing CSF NR	mNGS: sensitivity = 92.6%, specificity = 88.7%; Conventional microbiological tests: NR	NR	Bacterial, viral, fungal, tuberculosis, and parasitic EBV was the most common pathogen detected by CSF mNGS, found in 52.5% of samples, followed by CMV, John Cunningham virus (JCV), Torque teno virus (TTV), *Cryptococcus neoformans, Toxoplasma gondii*, and *Mycobacterium tuberculosis*	The high sensitivity of mNGS for detecting CNS viral infections like EBV, CMV, and JCV suggests that with further refinement, mNGS could potentially replace traditional viral detection tests for diagnosing these infections	It's challenging to fully eliminate the risk of unnecessary diagnostic investigations and treatments when using mNGS	21
Zou 2023 [53], China Retrospective Cohort Study, 41 mo (January 2019**—**June 2022)	Of 173 participants (140 living with HIV/AIDS, and 33 without HIV), 113 had infectious meningoencephalitis. The mean age of these patients was 39 (range 32–52) years, and 18% were female	DNA testing CSF CSF culture, PCR, and specific antibody tests not reported	NR	mNGS versus CMTs: sensitivity = 76.5%, specificity = 23.3%	Bacterial, viral, fungal, tuberculosis, and parasitic In patients living with HIV, EBV (33.6%) was the most common pathogen detected by mNGS, followed by CMV (20.7%) and Torque teno virus (TTV) (13.8%). In non-HIV patients, *Streptococcus* spp (18.2%), EBV (12.1%), and *Mycobacterium tuberculosis* (9.1%) were the top pathogens identified	mNGS detected infectious meningoencephalitis significantly more often than conventional methods: bacterial infections (12.4% vs 0.9%, *P* < .001), *Mycobacterium tuberculosis* (21.2% vs 2.7%, *P* < .001), and fungal infections (21.2% vs 11.5%, *P* = .048).	Identifying the pathogen should integrate clinical manifestations with results from both mNGS and conventional methods. However, interpretation of mNGS findings relies on clinician expertise, which may introduce some bias	20

Abbreviations: AFB, acid-fast bacillus stain; CMT, conventional microbiological test; CNS, central nervous system; CSF, cerebral spinal fluid; CMV, cytomegalovirus; D&B, Downs and Black; EBV, Epstein-Barr virus; ICU, intensive care unit; IQR, interquartile range; HSV, herpes simplex virus; mNGS, metagenomic next-generation sequencing; NR, not reported; PCR, polymerase chain reaction; SD, standard deviation; VZV, varicella-zoster virus.

All 34 studies evaluated the proportion of positive results between mNGS and CMTs [20–53]. These 34 papers reported a higher positive rate of bacteria, *Mycobacterium tuberculosis* (TB), fungi, virus, and parasites in mNGS than culture and CMTs [20–53]. The positive rate of mNGS varied across studies, with positive rates ranging from 43.5% to 93.5% for infectious meningoencephalitis when compared to clinical diagnosis [20–26, 28, 30–33, 35–37, 39, 40, 42, 43, 45–52]. The positive rate from CMTs varied from 10.0% to 67.5% for infectious meningoencephalitis when compared to clinical diagnosis [20, 21, 23, 25, 30, 32, 40, 42, 43, 45–49].

Of the 34 studies, various types of infectious meningoencephalitis were evaluated [20–53]. These included specific cases of TB (7 studies) [21, 30, 33, 39, 41, 45, 46]; bacterial, viral, fungal, and TB (9 studies) [22, 26, 32, 34, 36, 42, 44, 48, 50]; and bacterial, viral, fungal, TB, and parasitic (8 studies) [20, 23, 31, 37, 47, 49, 52, 53]. Additionally, fungal infections involving *Cryptococcus neoformans* were noted in 2 studies [25, 38]. Several studies investigated multiple etiologies, highlighting the complexity and varied nature of infectious meningoencephalitis [24, 27–29, 35, 40, 43, 51].

Ten studies evaluated the correlation of CSF abnormalities on the diagnostic yield of mNGS [20, 32, 35, 36, 41–43, 45, 48, 49]. Lu et al [35], Yuan et al [48], and Zhang et al 2020 [49] found higher white blood cell (WBC) counts correlated with increased mNGS detection rates. However, Wilson et al [43] and Benoit et al [20] found the opposite: higher WBC counts correlated with decreased mNGS sensitivity. Both Pan et al [36] and Wang et al 2022 [42] noted that pathogens causing low CSF glucose or high protein levels were more likely detected by mNGS. Concurrently, Wang et al 2019 [41] and Yan et al 2020 [45] observed significant correlations between glucose levels and mNGS detection rates, and higher CSF protein levels with mNGS positivity. Lin et al 2023 reported a decrease in CSF lymphocytes and glucose ratio with accurate TB meningitis (TBM) identification [32].

Regarding the quality assessment scores, 20 studies were considered good (>18 of 28 possible points) [20, 21, 23, 26–32, 35–38, 43, 48–50, 52, 53] per the Downs and Black quality tool and 13 studies were considered fair (15–18 points) [23, 25, 26, 34, 35, 40–43, 45–47, 49], indicating evidence to support the use of mNGS in the diagnosis of infectious meningoencephalitis. Only 1 study was considered of low quality (<15 points) [51].

### Diagnostic Accuracy

Overall, 23 studies, including 1660 patients who had meningoencephalitis, evaluated results of mNGS against the clinical diagnosis and were included in the meta-analysis [20–26, 30–33, 35, 36, 40, 42, 44–46, 48–52]. The meta-analysis results for mNGS revealed a pooled sensitivity of 0.70 (95% confidence interval [CI], .67–.72) across all 23 studies with a pooled specificity at 0.93 (95% CI, .92–.94). The DOR for mNGS stood at 26.7 (95% CI, 10.4–68.8). In a stratified analysis with 10 studies [20, 23, 25, 30, 32, 40, 42, 46, 48, 49] evaluating the performance of CMTs against the clinical diagnosis, CMTs exhibited a pooled sensitivity of 0.37 (95% CI, .34–.41) with a pooled specificity of 0.95 (95% CI, .94–.96). The DOR for CMTs was 12.2 (95% CI, 3.2–47.0; Table 2). We included forest plots showing the sensitivity and specificity of both mNGS and CMTs for detecting infectious meningoencephalitis from each study (Supplementary Appendices 5 and 6, respectively). The results of the meta-analysis were homogeneous for both analyses: the analysis comparing mNGS with clinical diagnosis (heterogeneity *P* = .39, *I*^2^ = 5%), and the analysis comparing CMTs with clinical diagnosis (heterogeneity *P* = .43, *I*^2^ = 1%).

**Table 2.** Diagnostic Accuracy of Metagenomic Next-generation Sequencing (mNGS) and the Conventional Microbiological Tests (CMTs) in Detecting Patients With Infectious Meningoencephalitis^a^

	Diagnostic Accuracy mNGS			Diagnostic Accuracy CMTs		
First Authors and Year	Sensitivity (95% CI)	Specificity (95% CI)	DOR (95% CI)	Sensitivity (95%)	Specificity (95%)	DOR (95% CI)
Benoit^b^ 2024 [20]	0.63 (.56–.69)	0.995 (.99–.998)	359.6 (136.8–945.5)	0.46 (0.40–0.53)	0.94 (0.92–0.96)	13.7 (9.3–20.1)
Chen 2022 [21]	0.84 (.75–.90)	0.996 (.96–1.0)	1320.4 (77.6–22456.4)	-	-	-
Chen 2024 [22]	0.44 (.34–.54)	0.52 (.40–.64)	0.9 (.5–1.6)	-	-	-
Deng 2023 [23]	0.64 (.47–.77)	0.65 (.39–.85)	3.3 (.9–12.4)	0.37 (0.21–0.56)	0.23 (0.11–0.43)	0.17 (.05–.6)
Fu 2024 [24]	0.86 (.70–.94)	0.44 (.24–.67)	4.8 (1.2–19.2)	-	-	-
Gan 2022 [25]	0.93 (.81–.97)	0.96 (.92–.98)	295.4 (77.2–1130.8)	0.63 (0.49–0.75)	0.997 (0.97–1.0)	500.7 (29.3–8558.4)
Gu 2022 [26]	0.65 (.50–.78)	0.71 (.53–.84)	4.6 (1.6–12.8)	-	-	-
Lin 2021 [30]	0.59 (.42–.73)	0.97 (.77–.997)	46.7 (2.6–841.8)	0.39 (0.24–0.55)	0.97 (0.77–0.997)	20.7 (1.1–374.6)
Lin AJTR 2023 [31]	0.97 (.87–.99)	0.88 (.76–.94)	217.0 (35.1–1341.7)	-	-	-
Lin DMID 2023 [32]	0.68 (.39–.88)	0.95 (.91–.97)	40.5 (9.7–168.6)	0.14 (0.03–0.42)	0.997 (0.97–1.0)	51.6 (2.0–1354.1)
Lin 2024 [33]	0.77 (.51–.91)	0.97 (.75–.997)	95.3 (4.5–2037.5)	-	-	-
Lu 2024 [35]	0.91 (.85–.95)	0.90 (.81–.95)	98.5 (34.5–280.6)	-	-	-
Pan 2023 [36]	0.79 (.57–.91)	0.96 (.83–.99)	81.9 (11.8–569.6)	-	-	-
Tian 2024 [40]	0.63 (.52–.74)	0.98 (.92–.995)	83.8 (15.4–455.1)	0.31 (0.21–0.42)	0.993 (0.94–0.999)	64.8 (3.8–1097.5)
Wang 2022 [42]	0.55 (.38–.71)	0.59 (.32–.82)	1.7 (.4–7.0)	0.33 (0.19–0.50)	0.86 (0.57–0.97)	3.1 (.5–20.1)
Xing 2020 [44]	0.28 (.17–.42)	0.96 (.92–.98)	9.7 (3.5–26.8)	-	-	-
Yan 2020 [45]	0.99 (.89–.999)	0.97 (.73–.996)	2079.0 (39.3–110006.0)	-	-	-
Yu 2021 [46]	0.44 (.26–.63)	0.97 (.75–.997)	22.6 (1.2–423.4)	0.35 (0.20–0.55)	0.97 (0.75–0.997)	15.9 (.8–301.0)
Yuan 2024 [48]	0.69 (.56–.79)	0.82 (.69–.91)	10.1 (4.1–25.0)	0.27 (0.18–0.39)	0.95 (0.85–0.98)	6.7 (1.7–26.8)
Zhang 2020 [49]	0.69 (.59–.78)	0.98 (.92–.995)	105.1 (19.6–563.2)	0.22 (0.15–0.32)	0.99 (0.93–0.999)	40.1 (2.4–677.5)
Zhang 2024 [50]	0.62 (.49–.72)	0.87 (.69–.95)	10.2 (3.0–35.0)	-	-	-
Zhao 2020 [51]	0.59 (.48–.69)	0.02 (.002–.19)	0.03 (.002–.57)	-	-	-
Zhu 2022 [52]	0.92 (.82–.97)	0.89 (.85–.91)	87.4 (31.7–240.8)	-	-	-
All studies	Pooled sensitivity	Pooled specificity	DOR (95% CI)	Pooled sensitivity	Pooled specificity	DOR (95% CI)
All studies	0.70 (0.67–0.72)	0.93 (0.92–0.94)	26.7 (10.4–68.8)	0.37 (0.34–0.41)	0.95 (0.94–0.96)	12.2 (3.2–47.0)

Abbreviations: CI, confidence interval; DOR, diagnostic odds ratio.

^a^The raw data of the included studies used in the meta-analysis are in Supplementary Appendix 3.

^b^The Wilson et al study [43] was not included in the meta-analysis because of patients’ overlap in the Benoit et al study [20].

We also performed a stratified analysis with 7 studies investigating TBM (N = 636) and evaluating the results of mNGS against the clinical diagnosis [21, 30, 33, 42, 44–46]. We sought to include data from definite cases of TBM [21, 33, 44, 45], as defined by Marais et al [54], but we also included studies in which we could not separate definite from probable TBM [30, 42, 46]. The meta-analysis results for mNGS revealed a pooled sensitivity of 0.67 (95% CI, .61–.72) and a pooled specificity at 0.97 (95% CI, .95–.99). The DOR for mNGS stood at 43.5 (95% CI, 7.4–256.6). In a stratified analysis with 3 studies [30, 42, 46] evaluating the performance of CMTs against the clinical diagnosis, CMTs exhibited a pooled sensitivity of 0.35 (95% CI, .34–.45) with a pooled specificity of 0.98 (95% CI, .87–.996). The DOR for CMTs was 6.9 (95% CI, 1.7–27.6; Table 3).

**Table 3.** Diagnostic Accuracy of Metagenomic Next-generation Sequencing (mNGS) and the Conventional Microbiological Tests (CMTs) in Detecting Patients With Tuberculosis Meningoencephalitis^a^

	Diagnostic Accuracy mNGS			Diagnostic Accuracy CMTs		
First Authors and Year	Sensitivity (95% CI)	Specificity (95% CI)	DOR (95% CI)	Sensitivity (95%)	Specificity (95%)	DOR (95% CI)
Chen 2022 [21]	0.84 (.75–.90)	0.996 (.96–1.0)	1320.4 (77.6–22456.4)	-	-	-
Lin 2021 [30]	0.59 (.42–.73)	0.97 (.77–.997)	46.7 (2.6–841.8)	0.39 (0.24–0.55)	0.97 (0.77–0.997)	20.7 (1.1–374.6)
Lin 2024 [33]	0.77 (.51–.91)	0.97 (.75–.997)	95.3 (4.5–2037.5)	-	-	-
Wang 2022 [42]	0.55 (.38–.71)	0.59 (.32–.82)	1.7 (.4–7.0)	0.33 (0.19–0.50)	0.86 (0.57–0.97)	3.1 (.5–20.1)
Xing 2020 [44]	0.28 (.17–.42)	0.96 (.92–.98)	9.7 (3.5–26.8)	-	-	-
Yan 2020 [45]	0.99 (.89–.999)	0.97 (.73–.996)	2079.0 (39.3–110006.0)	-	-	-
Yu 2021 [46]	0.44 (.26–.63)	0.97 (.75–.997)	22.6 (1.2–423.4)	0.35 (0.20–0.55)	0.97 (0.75–0.997)	15.9 (.8–301.0)
All studies	Pooled sensitivity	Pooled specificity	DOR (95% CI)	Pooled sensitivity	Pooled specificity	DOR (95% CI)
All studies	0.67 (0.61–0.72)	0.97 (0.95–0.99)	43.5 (7.4–256.6)	0.35 (0.26–0.46)	0.98 (0.87–0.996)	6.9 (1.7–27.6)

Abbreviations: CI, confidence interval; DOR, diagnostic odds ratio.

^a^The raw data of the included studies used in the meta-analysis are in Supplementary Appendix 4.

### Publication Bias

The Egger's regression test did not indicate publication bias among the studies included in the meta-analysis (*P* = .19).

## DISCUSSION

This systematic literature review and meta-analysis provides a comprehensive evaluation of mNGS as a diagnostic tool for infectious meningoencephalitis by comparing it to CMTs. The results indicate that mNGS has significantly higher sensitivity than CMTs, with pooled sensitivity rates of 70% versus 37%, respectively. This higher sensitivity underscores mNGS's ability to detect a broader spectrum of pathogens, including rare and difficult-to-culture organisms, which traditional methods might miss. Moreover, the pooled specificity of mNGS (93%) is comparable to that of CMTs (95%), suggesting that mNGS maintains a high level of accuracy in identifying true negatives.

The analysis also revealed that mNGS's DOR is higher than that of CMTs, indicating superior diagnostic performance. Specifically, mNGS had a DOR of 26.7 compared to 12.2 for CMTs. For TBM, the sensitivity of mNGS was 67% with a specificity of 97%, demonstrating its greater effectiveness in diagnosing this condition compared to CMTs, which had a sensitivity of 35% and a specificity of 98%. These findings suggest that mNGS is a valuable tool for enhancing the diagnosis of infectious meningoencephalitis, particularly for complex cases where traditional methods fall short.

Another important consideration is that the utility of mNGS, like CMTs, is significantly impacted by when the testing occurs in the diagnostic workup [3, 5, 55]. For example, if the test is done early in the infectious meningoencephalitis workup, its impact is likely to be more significant [3, 5]. However, the test is often not requested at an early stage, which may account for some of the variability in positive rates observed across studies [20, 21, 23, 25, 30, 32, 40, 42, 43, 45–49].

Several studies within the review highlighted specific correlations between CSF parameters and mNGS detection rate [35, 36, 42, 45, 48, 49]. For instance, higher WBC counts, and CSF protein levels were associated with increased mNGS positivity. This correlation was evident in studies by Lu et al [35], Yan et al [45], Yuan et al [48], and Zhang et al 2020 [49], which noted that elevated CSF protein and WBC counts significantly predicted mNGS positive results. However, Wilson et al [43] and Benoit et al [20] showed a decreased sensitivity for mNGS with particularly high CSF WBC counts. Studies by Pan et al [36] and Wang et al 2022 [42] found that low CSF glucose and high protein levels were more likely to yield positive mNGS results, although this correlation may be attenuated in immunocompromised individuals [56]. These findings underscore the importance of considering CSF characteristics when interpreting mNGS results because they can influence the diagnostic yield. Although it would also be of interest to compare the efficacy of mNGS using distinct sample types, such as comparing CSF to serum, almost all studies only investigated CSF.

Standardizing protocols and quality control measures for mNGS is important to ensure its effectiveness [3, 15, 57]. Given the nuanced interpretation required for mNGS results, whether positive or negative, it is essential for hospitals to implement strict guidelines for authorizing test orders. This responsibility should be entrusted to infectious disease physicians, neurologists, or experienced microbiologists who have the expertise to interpret mNGS data accurately. Additionally, to maximize the utility of mNGS and reduce the risk of misinterpretation, hospitals might restrict its use to cases where all CMTs have been thoroughly conducted and yielded negative results. However, performing mNGS later in the diagnostic process may result in a lower impact, and specimens from the initial sample may need to be stored for later testing. When detected microbial DNA or RNA is below a predetermined threshold, microbiology laboratories should consider withholding such findings to prevent confusion among healthcare providers.

Despite the promising results, several limitations this systematic review should be acknowledged. The variability in the study designs, populations, mNGS library preparation methodologies, bioinformatics pipelines, and statistical criteria for differentiating between a “true” infection and an environmental or laboratory contaminant across the included studies may affect the generalizability of the findings [58]. The predominance of studies conducted in China (27 of 34) might introduce a geographic bias, limiting the applicability of the results to other regions with different pathogen prevalence and healthcare practices [21–26, 30–36, 39–42, 44–53]. Additionally, the retrospective nature of most studies (22 of 34) could introduce recall bias and limit the ability to draw casual inferences [20, 21, 25, 26, 31, 32, 34–36, 38, 39, 41, 42, 45–48, 50–53]. Clinical adjudication was performed by the authors of the individual studies, not by the current authors, and our review did not specify how undiagnosed cases were handled—a significant limitation considering the high proportion of undiagnosed cases in meningoencephalitis literature. This variability, combined with the inclusion of controversial organisms (eg, Epstein Barr virus, cytomegalovirus), highlights the need for clearer adjudication processes. These factors, along with differences in assay methodologies, impact diagnostic performance metrics, including the DOR. The wide range of DOR values and large CIs reflect the influence of these variables, making it difficult to draw definitive conclusions on mNGS's diagnostic accuracy across diverse settings. A more detailed exploration of how assay variables and clinical settings influence DOR would have strengthened the interpretation of these findings. Additionally, the lack of consistent detection of Japanese encephalitis virus in the studies may point to limitations in mNGS for detecting such pathogens, which may be better identified through serologic methods, especially in regions where Japanese encephalitis virus is endemic. Furthermore, although the meta-analysis demonstrated the diagnostic superiority of mNGS, the clinical impact in terms of patient outcomes and cost-effectiveness remains less clear. The integration of mNGS into routine clinical practice also necessitates considerations of its accessibility, cost, and the need for specialized training for laboratory personnel [3, 57]. Additionally, the potential for contamination and the presence of background noise in mNGS data can lead to false positives, complicating the interpretation of results [3, 59–61]. This issue underscores the need for stringent quality control measures and robust bioinformatics pipelines to ensure the accuracy of pathogen identification [58]. The high cost and resource intensity associated with mNGS might also limit its widespread adoption, particularly in resource-limited settings [62]. Moreover, our review did not sufficiently address the turnaround time for mNGS compared to CMTs. Although some mNGS laboratories are able to offer increasingly rapid results (<48 hours), the actual time from sample collection to diagnostic reporting can vary significantly depending on the laboratory infrastructure and expertise [5].

In conclusion, this systematic literature review and meta-analysis affirm the diagnostic advantages of mNGS over CMTs for infectious meningoencephalitis, including tuberculosis meningoencephalitis, highlighting its higher sensitivity and comparable specificity. The ability of mNGS to detect a wide array of pathogens rapidly positions it as a transformative tool in the clinical diagnosis of this complex condition. However, addressing the noted limitations through future research will be important to fully realize its potential and ensure its effective implementation in diverse clinical settings.

## Supplementary Material

ofaf274_Supplementary_Data

## Supplementary Data

Supplementary materials are available at *Open Forum Infectious Diseases* online. Consisting of data provided by the authors to benefit the reader, the posted materials are not copyedited and are the sole responsibility of the authors, so questions or comments should be addressed to the corresponding author.
