# Supplementary material for: Metagenomic Next-generation Sequencing in Patients With Infectious Meningoencephalitis: A Comprehensive Systematic Literature Review and Meta-analysis
Source: Open Forum Infect Dis. 2025 May 9;12(5):ofaf274. doi: 10.1093/ofid/ofaf274 (PMC12117655; doi:10.1093/ofid/ofaf274)

**Metagenomic next-generation sequencing in patients with infectious meningoencephalitis: A comprehensive systematic literature review and meta-analysis**

Supplemental Material

# Pedro S. Marra*^1^, Alexandre R. Marra^2,3^, Eileen Chen^1^, Takaaki Kobayashi^3^, Patrícia Deffune Celeghini^2^, Maria Celidonio Gutfreund^2^, Isabele Pardo^2^, Gabriel O.V. Lopes^2^, Mariana Kim Hsieh^4^, Nicole A. Boodhoo^5^, Daniel Fu^6^, Michael A. Torres-Espinosa^1^, Yimeng Li^1^, Rodrigo Octávio Deliberato^7,8^, Sulwan Mujahid A. Algain^9^, Jorge L. Salinas^9^, Michael B. Edmond^10^, Deyvid Emanuel Amgarten^2^, Fernanda de Mello Malta^2^, Nathalia Villa dos Santos^2^, João Renato Rebello Pinho^2,11^, Martineau Louine^12^, Michael R. Wilson^12^.

1. School of Medicine, University of California San Francisco, San Francisco, CA, USA
2. Faculdade Israelita de Ciências da Saúde Albert Einstein, Hospital Israelita Albert Einstein, São Paulo, SP, Brazil
3. University of Iowa Health Care, Department of Internal Medicine, Iowa City, IA, USA
4. Program of Hospital Epidemiology, University of Iowa Health Care, Iowa City, IA, USA
5. University of Iowa College of Public Health, Department of Epidemiology, Iowa City, IA, USA
6. Pritzker School of Medicine, University of Chicago, Chicago, IL, USA
7. Department of Biostatistics, Health Informatics and Data Science, University of Cincinnati College of Medicine, Cincinnati, OH, USA
8. Biomedical Informatics Division, Cincinnati Children’s Hospital Medical Center, Cincinnati, OH, USA
9. Division of Infectious Diseases & Geographic Medicine, Stanford University, Stanford, CA, USA
10. Department of Medicine, West Virginia University School of Medicine, Morgantown, WV, USA
11. LIM03/07, Hospital das Clínicas da Faculdade de Medicina da Universidade de São Paulo, São Paulo, SP, Brazil
12. Weill Institute of Neurosciences, Department of Neurology, University of California San Francisco, San Francisco, CA, USA.

**Contents**

1. **Cover Page** …………………..……………………................................................ 1
2. **Contents** …………………………..………............................................................ 2
3. **Supplementary Appendix 1**. Search terms and strategy ………………………. 3
4. **Supplementary Appendix 2**: Standardized Data Abstract Form...…………...… 6
5. **Supplementary Appendix 3:** Raw data of included studies with clinical diagnosis for mNGS and for the conventional microbiological tests (CMTs)…………….............................................................................................. 23
6. **Supplementary Appendix 4:** Raw data of included tuberculosis meningoencephalitis studies with clinical diagnosis for mNGS and for the conventional microbiological tests (CMTs)………………………………………… 25
7. **Supplementary Appendix 5:** Forest plot showing the sensitivity and specificity of metagenomic next-generation sequencing (mNGS) for detecting infectious meningoencephalitis in patients……………………………………………………. 26
8. **Supplementary Appendix 6:** Forest plot showing the sensitivity and specificity of conventional microbiological tests (CMTs) for detecting infectious meningoencephalitis in patients……………………………………………………. 27

**Supplementary Appendix 1.** Search terms and strategies

Pubmed:

- ("Meningoencephalitis"[Mesh] OR "Meningoencephalitis"[tw] OR "Meningitis"[tw] OR "meningitis"[mesh] OR "Encephalitis"[tw] OR "encephalitis"[mesh]) AND ("Next-generation sequencing"[tw] OR "High-Throughput Nucleotide Sequencing"[Mesh] OR "mNGS"[tw]) **NOT ("interview"[Publication Type] OR "case reports"[Publication Type])**
- Results: 488

Embase:

- ('meningoencephalitis'/exp OR 'meningoencephalitis':ti,ab,kw,de,dn,df,mn,tn OR 'meningitis':ti,ab,kw,de,dn,df,mn,tn OR 'meningitis'/exp OR 'encephalitis':ti,ab,kw,de,dn,df,mn,tn OR 'encephalitis'/exp) AND ('next-generation sequencing':ti,ab,kw,de,dn,df,mn,tn OR 'high throughput sequencing'/exp OR 'mngs':ti,ab,kw,de,dn,df,mn,tn)

Results: 695 (after limiting to Embase only)

Web of Science:

- ("Meningoencephalitis" OR "Meningitis" OR "Encephalitis") and ("Next-generation sequencing" OR "mNGS")

Filters: Topic Search

Results: 672

CINAHL:

- ((MH "Meningoencephalitis") OR "meningoencephalitis" OR (MH "Meningitis+") OR (MH "Encephalitis+") OR "meningitis" OR "encephalitis" ) AND ( "Next-generation sequencing" OR "mNGS" )

Filters: none

Results: 109

Cochrane:


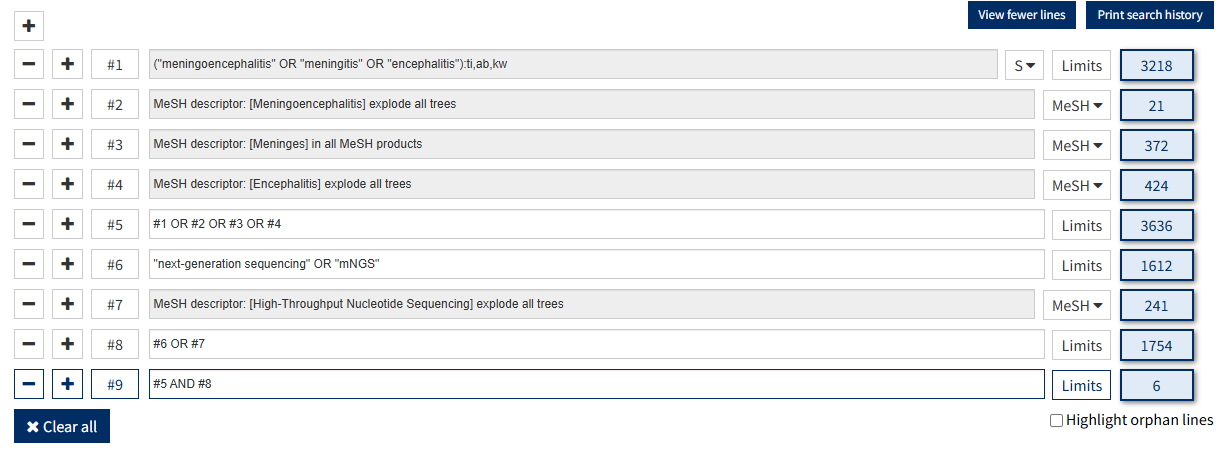


Results: 6

ClinicalTrials.gov:


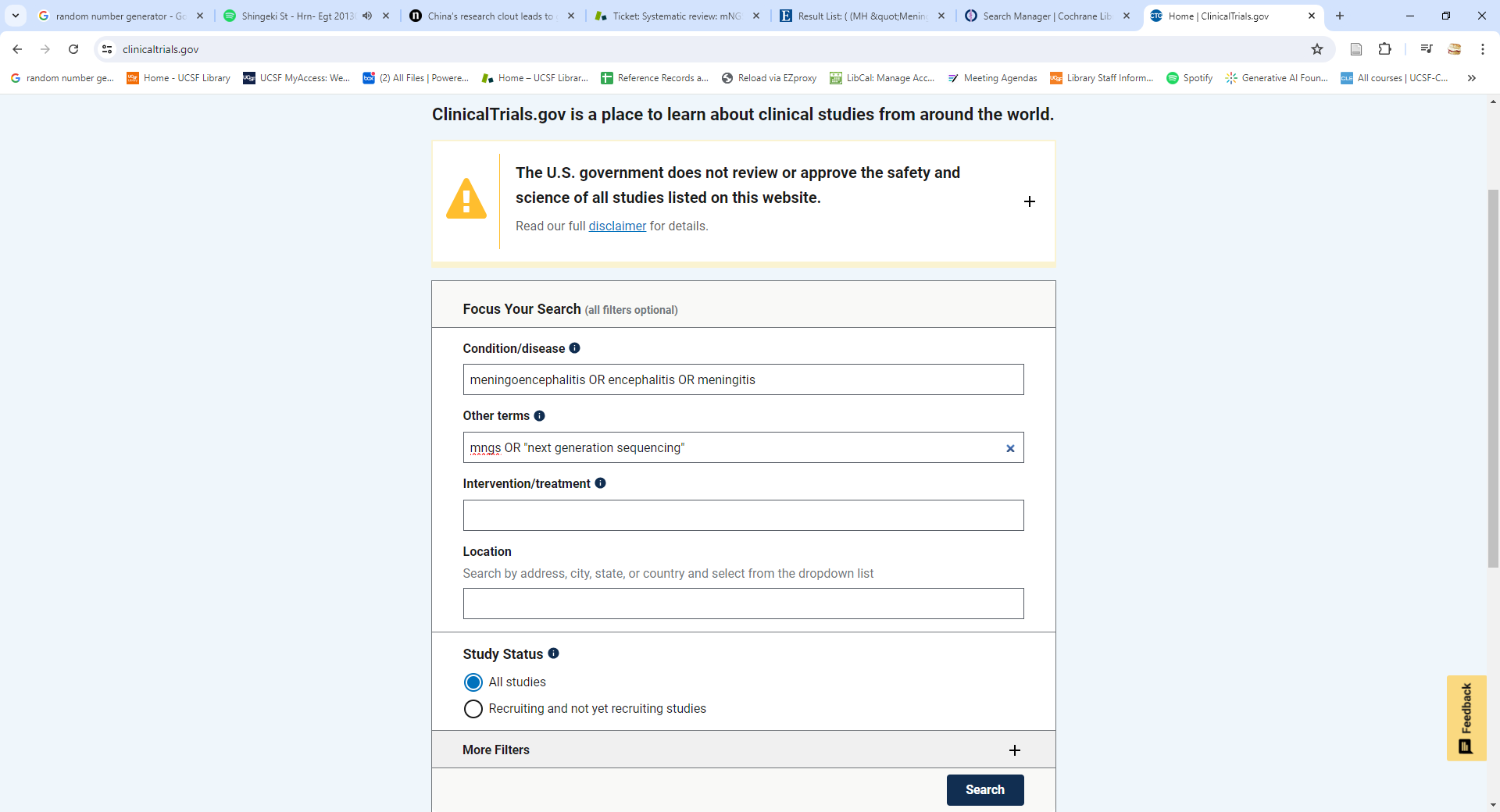


Results: 8

**Supplementary Appendix 2**: Standardized Data Abstract Form

**DATA ABSTRACT FORM – Metagenomic Next-Generation Sequencing in Patients with Meningoencephalitis: A Comprehensive Systematic Review and Meta-Analysis**

1. First author last name:_____________________________________________________
2. Publication year:__________________________________________________________
3. Study location (City, State, Country):__________________________________________
4. Reviewer’s initials:_________________________________________________________

**Part 1.**

Basic Inclusion/Exclusion Criteria:

1. Did the study evaluate **metagenomic next-generation sequencing (mNGS)**? □ Yes □No (if no, exclude it)
2. Did the study evaluate **mNGS vs. other diagnostic methods (culture or conventional microbiological tests (e.g., serologic test, antigenic test, or nucleic acid amplification [PCR] test):__________________________[write here])**? □ Yes □No (*if no, we will not exclude it. We will keep this information to make a decision in the future. It will depend on the number of studies that we have found*)
3. Did the study include patients with meningoencephalitis, meningitis, and/or encephalitis**?** (if no, exclude it) □ Yes □No
4. Duration of study:___________(in weeks or months)
5. Do you believe this study should be excluded? □ Yes □No
6. If yes, why? _______________________________________________________________

**Part 2.**

Exposure and Outcomes Assessment (Check more than one if it is necessary)

1. Where did the study take place?

□ An academic medical center

□ A community hospital

□ A nursing homes

□ Other: ______________________________________

1. Which type is this study design?

□ Retrospective Cohort study

□ Prospective Cohort study

□ Case-control study

□ Randomized controlled trial

□ Quasi-experimental study

□ Transversal study

1. Was the study performed in more than one hospital? If yes, please add the number of hospitals

□Yes (If yes, please add the # of hospitals) ___ □ No

1. Which type of mNGS test? (you can circle more than one)

□ DNA and RNA testing

□ DNA testing

□ RNA testing

□ Karius test (This is a DNA testing but qualitative/quantitative – you can circle more than one)

□Other test (which name:____________________)

□ Not reported

1. What type is the studied sample?

□ blood

□ CSF

□ respiratory secretions

□ stool

□biopsy

□ other:__________________________________________________

6. Did the study evaluate **people with immunocompromised conditions**? □ Yes □No

7.How did the study define an **immunocompromised/immunosuppressed** person?

□ Medications: corticosteroids, chemotherapy or other immunosuppressive medications □ HIV

□ Solid organ transplant □ Hematopoietic stem cell transplant □ Thalassemia

□ Active cancer (current cancer or in treatment or received diagnosis within last 12 months)

□ Others: _______________________________________

8. **Population characteristics of patients with meningoencephalitis/meningitis/encephalitis** (Fill out only IF the information is available on the papers)

|  | **Infectious disease** | **Non-Infectious disease** |
| --- | --- | --- |
| Total number and % |  |  |
| Age (Mean [SD] or Median [IQR]):  Include number of pediatric patients if any |  |  |
| % of female |  |  |
| Length of hospital stay, days |  |  |
| Type of antibiotics |  |  |
| Duration of antibiotics treatment, days |  |  |

9. **Diagnostic test characteristics of patients with meningoencephalitis/meningitis/encephalitis** (Fill out only IF the information is available on the papers)

|  | **mNGS** | **culture** | **Other test (___________)** |
| --- | --- | --- | --- |
| Total number |  |  |  |
| Detection rate of rare pathogens (%) |  |  |  |
| Name of the top 3 pathogens most identified in each category:   - Bacteria - Fungus - Virus - Mycobacteria |  |  |  |
| Time spent on diagnosis (days) |  |  |  |
| What was the earliest test to make the diagnosis? |  |  |  |
| Length of hospital stay, days |  |  |  |
| Duration of antibiotics treatment, days |  |  |  |
| Cost (mean or median) |  |  |  |

11. How did CSF compare to other sample types (like plasma) to diagnose meningoencephalitis, meningitis, and/or encephalitis? Did it have higher or lower sensitivity/specificity than other sample types?

__________________________________________________________________________________________________________________________________________________________________________________________________________________________________________________________________________________________________________________________________________________________

12. Were there advantages of mNGS compared to traditional pathogen diagnostic methods for patients with meningoencephalitis: □ Yes □No (Describe the conclusions of this advantage or not):_______________________________________________________________________________________________________________________________________________________________________________________________________________________________________________________________________________________________________________________________________________________________________________________________________________________________________________________________________________________________________________________________________________________________________________________________

13. What kind of barriers or challenges did the authors mention when using mNGS or implementing it in the clinical setting?: ______________________________________________________________________________________________________________________________________________________________________________________________________________________________________________________________________________________________________________________________________________________________________________________________________________________________________________________________________________________________________________________________________________________________________________________________________________________________________________________________________

14. Did the study review the reasoning behind NGS testing? □ Yes □No

If yes, which reasons were given? Please share any quantitative data gathered by the study.

For example, a study might show that among 100 NGS tests ordered, 50% of them were ordered due to a concern for fungal infection, 25% for sepsis, and 25% for recurrent fever.

_____________________________________________________________________________________________________________________________________________________________________________________________________________________________________________________________________________________________________________________________________

15. Did the study investigate the clinical impact of mNGS (e.g. whether there was a change in management or treatment of the patient following mNGS results, etc.)?  □ Yes □No

Please share the kind of impact and any quantitative data gathered by the study.

For example, a study might show that mNGS had a positive impact in the analysis of 100 cases (10%), such as through new diagnosis in 80 cases (5%).

______________________________________________________________________________________________________________________________________________________________________________________________________________________________________________________________________________________________________________________________________

16. Did the study attempt to increase sensitivity and/or specificity of mNGS by adding extra steps to its normal pipeline or coupling it with another method? If yes, what did researchers do?

For example, a study might remove human background DNA to improve detection of pathogen DNA or a study might couple mNGS with a machine learning classifier to help improve diagnostic accuracy.

___________________________________________________________________________________________________________________________________________________________________________________________________________________________________________________________________________________________________________________________________

17. Did the patients who tested positive with mNGS have more CSF abnormalities (like high protein or low glucose) compared to those for whom mNGS did not find a diagnosis? Please specify the differences.

__________________________________________________________________________________________________________________________________________________________________________________________________________________________________________________________________________________________________________________________________

**Part 3**. Unadjusted and adjusted associations

1. Raw numbers: Please fill raw data for the following tables if available. Only include measures of effect if they are listed in the manuscript. Please make sure that each sample is equivalent to one patient.

**Table A: Main Association of Interest – mNGS and final clinical diagnosis [reference standard*] (total number)**

***For the clinical diagnosis or “clinical adjudication”, experts can consider as a r*eference standard some microbiology methods as a culture, a serology test, antigenic test, etc. Please specify it when filling out the table if possible (all methods or culture or serology test, etc.)***

Clinical diagnosis -

Clinical diagnosis +

|  |  |
| --- | --- |
|  |  |

Diagnosis from mNGS +

Diagnosis from mNGS -

**Diagnostic performance:**

Sensitivity:

Specificity:

Positive predictive value (PPV):

Negative predictive value (NPV):

**Table B: Main Association of Interest – Conventional microbiological test (CMT) [reference standard*] and final clinical diagnosis (total number)**

****Reference standard can be a culture, a serology test, antigenic test, etc. Please specify it when filling out the table if possible (all methods or culture or serology test, etc.)***

|  |  |
| --- | --- |
|  |  |

Clinical diagnosis -

Clinical diagnosis +

CMT +

CMT -

**Diagnostic performance:**

Sensitivity:

Specificity:

Positive predictive value (PPV):

Negative predictive value (NPV):

**Table C: Main Association of Interest – mNGS and CMT for all patients with meningoencephalitis [reference standard*] (total number)**

****Reference standard can be a culture, a serology test, antigenic test, etc. Please specify it when filling out the table if possible (all methods or culture or serology test, etc.)***

CMT +

CMT -

|  |  |
| --- | --- |
|  |  |

Diagnosis from mNGS +

Diagnosis from mNGS -

**Diagnostic performance:**

Sensitivity:

Specificity:

Positive predictive value (PPV):

Negative predictive value (NPV):

**Table D: Main Association of Interest – mNGS and CMT for only immunocompetent patients with meningoencephalitis [reference standard*] (total number)**

****Reference standard can be a culture, a serology test, antigenic test, etc. Please specify it when filling out the table if possible (all methods or culture or serology test, etc.)***

CMT -

CMT +

|  |  |
| --- | --- |
|  |  |

Diagnosis from mNGS +

Diagnosis from mNGS -

**Diagnostic performance:**

Sensitivity:

Specificity:

Positive predictive value (PPV):

Negative predictive value (NPV):

**Table E: Main Association of Interest – mNGS and CMT for only immunocompromised patients with meningoencephalitis [reference standard*] (total number)**

****Reference standard can be a culture, a serology test, antigenic test, etc. Please specify it when filling out the table if possible (all methods or culture or serology test, etc.)***

CMT +

CMT -

|  |  |
| --- | --- |
|  |  |

Diagnosis from mNGS +

Diagnosis from mNGS -

**Diagnostic performance:**

Sensitivity:

Specificity:

Positive predictive value (PPV):

Negative predictive value (NPV):

***If the study investigated the diagnostic performance of mNGS in multiple types of samples (e.g. blood, CSF, etc.), please include another 2x2 table and fill out the information following the same format as Tables A-D.***

**Part 4.** Other references

Please look through the references. Are there other references that we should evaluate for the meta-analysis? If yes, please provide first author, journal and year _______________________________________________________________________________________________________________________________________________________________________________________________________________________________________________________________

**Part 5: Quality Assessment Tool:**

Adapted Downs and Black Tool:

1. Is the hypothesis/aim/objective of the study clearly described?

| yes | 1 |
| --- | --- |
| no | 0 |

2. Are the main outcomes to be measured clearly described in the Introduction or Methods section?

*If the main outcomes are first mentioned in the Results section, the question should be answered no.*

| yes | 1 |
| --- | --- |
| no | 0 |

3. Are the characteristics of the participants included in the study clearly described?

*In cohort and cross-sectional studies, inclusion and/or exclusion criteria should be given. In case-control studies, a case-definition and the sources for controls should be given.*

| yes | 1 |
| --- | --- |
| no | 0 |

4. Are the interventions of interest clearly described?

*Treatments and placebo (where relevant) that are to be compared should be clearly described.*

| yes | 1 |
| --- | --- |
| no | 0 |

5. Are the distributions of principal confounders in each group of subjects to be compared clearly described? *A list of principal confounders is provided.*

| yes | 2 |
| --- | --- |
| partially | 1 |
| no | 0 |

6. Are the main findings of the study clearly described?

*Simple outcome data (including denominators and numerators) should be reported for all major findings so that the reader can check the major analyses and conclusions. (This question does not cover statistical tests which are considered below).*

| yes | 1 |
| --- | --- |
| no | 0 |

7. Does the study provide estimates of the random variability in the data for the main outcomes?

*In non-normally distributed data, the inter-quartile range of results should be reported. In normally distributed data the standard error, standard deviation or confidence intervals should be reported. If the distribution of the data is not described, it must be assumed that the estimates used were appropriate and the question should be answered yes.*

| yes | 1 |
| --- | --- |
| no | 0 |

8. Have all important adverse events that may be a consequence of the intervention been reported?

*This should be answered yes if the study demonstrates that there was a comprehensive attempt to measure adverse events. (A list of possible adverse events is provided).*

| yes | 1 |
| --- | --- |
| no | 0 |

9. Have the characteristics of patients lost to follow-up been described?

*This should be answered yes where there were no losses to follow-up or where losses to follow-up were so small that findings would be unaffected by their inclusion. This should be answered nowhere a study does not report the number of patients lost to follow-up.*

| yes | 1 |
| --- | --- |
| no | 0 |

10. Have actual probability values been reported (e.g. 0.035 rather than <0.05) for the main outcomes except where the probability value is less than 0.001?

| yes | 1 |
| --- | --- |
| no | 0 |

External validity:

All the following criteria attempt to address the representativeness of the findings of the study and whether they may be generalized to the population from which the study subjects were derived.

11. Were the subjects asked to participate in the study representative of the entire population from which they were recruited?

*The study must identify the source population for patients and describe how the patients were selected. Patients would be representative if they comprised the entire source population, an unselected sample of consecutive patients, or a random sample. Random sampling is only feasible where a list of all members of the relevant population exists. Where a study does not report the proportion of the source population from which the patients are derived, the question should be answer as unable to determine.*

| yes | 1 |
| --- | --- |
| no | 0 |
| unable to determine | 0 |

12. Were those subjects who were prepared to participate representative of the entire population from which they were recruited?

*The proportion of those asked who agreed should be stated. Validation that the sample was representative would include demonstrating that the distribution of the main confounding factor was the same in the study sample and the source population.*

| yes | 1 |
| --- | --- |
| no | 0 |
| unable to determine | 0 |

13. Were the staff, places, and facilities where the patients were treated, representative of the treatment most patients receive?

*For the question to be answered yes, the study should demonstrate that the intervention was representative of that in use in the source population. The question should be answered no if, for example, the intervention was undertaken in a specialist centre unrepresentative of the hospitals most of the source population would attend.*

| yes | 1 |
| --- | --- |
| no | 0 |
| unable to determine | 0 |

Internal validity – bias

14. Was an attempt made to blind study subjects to the intervention they have received?

*For studies where the patients would have no way of knowing which intervention they received, this should be answered yes.*

| yes | 1 |
| --- | --- |
| no | 0 |
| unable to determine | 0 |

15. Was an attempt made to blind those measuring the main outcomes of the intervention?

| yes | 1 |
| --- | --- |
| no | 0 |
| unable to determine | 0 |

16. If any of the results of the study were based on “data dredging,” was this made clear?

*Any analyses that had not been planned at the outset of the study should be clearly indicated. If no retrospective unplanned subgroup analyses were reported, then answer yes.*

| yes | 1 |
| --- | --- |
| no | 0 |
| unable to determine | 0 |

17. In trials and cohort studies, do the analyses adjust for different lengths of follow-up of patients, or in case-control studies, is the period between the intervention and outcome the same for cases and controls?

*Where follow-up was the same for all study patients the answer should be yes. If different lengths of follow-up were adjusted by, for example, survival analysis, the answer should be yes. Studies in which differences in follow-up are ignored, the answer should be no.*

| yes | 1 |
| --- | --- |
| no | 0 |
| unable to determine | 0 |

18. Were the statistical tests used to assess the main outcomes appropriate?

*The statistical techniques must be appropriate to the data. For example, non-parametric methods should be used for small sample sizes. Where little statistical analysis has been undertaken but where there is no evidence of bias, the question should be answered yes. If the distribution of the data (normal or not) is not described it must be assumed that the estimates used were appropriate and the question should be answered yes.*

| yes | 1 |
| --- | --- |
| no | 0 |
| unable to determine | 0 |

19. Was compliance with the intervention/s reliable?

*Where there was noncompliance with the allocated treatment or where there was contamination of one group, the question should be answered no. For studies where the effect of any misclassification was likely to bias any association to the null, the question should be answered yes.*

| yes | 1 |
| --- | --- |
| no | 0 |
| unable to determine | 0 |

20. Were the main outcome measures used accurate (valid and reliable)?

*For studies where the outcome measures are clearly described, the question should be answered yes. For studies which refer to other work or that demonstrates the outcome measures are accurate, the question should be answered as yes.*

| yes | 1 |
| --- | --- |
| no | 0 |
| unable to determine | 0 |

Internal validity – confounding (selection bias)

21. Were the patients in different intervention groups (trials and cohort studies)/ cases and controls (case-control studies) recruited from the same population?

*For example, patients for all comparison groups should be selected from the same hospital. The question should be answered unable to determine for cohort and case-control studies where there is no information concerning the source of patients included in the study.*

| yes | 1 |
| --- | --- |
| no | 0 |
| unable to determine | 0 |

22. Were study subjects in different intervention groups (trials and cohort studies)/ cases and controls (case-control studies) recruited over the same period of time?

*For a study which does not specify the time period over which patients were recruited, the question should be answered as unable to determine.*

| yes | 1 |
| --- | --- |
| no | 0 |
| unable to determine | 0 |

23. Were study subjects randomised to intervention groups?

*Studies which state that subjects were randomized should be answered yes except when the method of randomization would not ensure random allocation. For example, alternate allocation would score no because it is predictable.*

| yes | 1 |
| --- | --- |
| no | 0 |
| unable to determine | 0 |

24. Was the randomized intervention assignment concealed from both patients and health care staff until recruitment was complete and irrevocable?

| yes | 1 |
| --- | --- |
| no | 0 |
| unable to determine | 0 |

25. Was there adequate adjustment for confounding in the analyses from which the main findings were drawn?

*This question should be answered as no for trials if: the main conclusions of the study were based on analyses of treatment rather than intention to treat; the distribution of known confounders in the different treatment groups was not described; or the distribution of known confounders differed between the treatment groups but was not considered in the analyses. In non-randomized studies, if the effect of the main confounders was not investigated or confounding was demonstrated but no adjustment was made in the final analyses, the question should be answered as no.*

| yes | 1 |
| --- | --- |
| no | 0 |
| unable to determine | 0 |

26. Were losses of patients to follow-up considered?

*If the number of patients lost to follow-up are not reported, the question should be answered as unable to determine. If the proportion lost to follow-up was too small to affect the main findings, the question should be answered as yes.*

| yes | 1 |
| --- | --- |
| no | 0 |
| unable to determine | 0 |

Power

27. Did the study perform calculations to determine sufficient power to detect a clinically important difference?

*Sample sizes have been calculated to detect a difference of x% and y%.*

| Yes | 1 |
| --- | --- |
| No | 0 |

**Total score: _____**

**Supplementary Appendix 3.** Raw data of included studies with clinical diagnosis

| Diagnostic  Test | First Author, Year | TP | FP | FN | TN |
| --- | --- | --- | --- | --- | --- |
| mNGS | Benoit 2024 | 135 | 4 | 79 | 949 |
|  | Chen 2022* | 74 | 0 | 14 | 128 |
|  | Chen 2024 | 39 | 30 | 50 | 33 |
|  | Deng 2023 | 23 | 4 | 13 | 8 |
|  | Fu 2024 | 27 | 9 | 4 | 7 |
|  | Gan 2022 | 43 | 6 | 3 | 154 |
|  | Gu 2022 | 25 | 8 | 13 | 20 |
|  | Lin 2021 | 20 | 0 | 14 | 16 |
|  | Lin 2023 | 45 | 6 | 1 | 46 |
|  | Lin 2023 | 7 | 9 | 3 | 179 |
|  | Lin 2024* | 11 | 0 | 3 | 14 |
|  | Lu 2024 | 100 | 6 | 9 | 60 |
|  | Pan 2023 | 16 | 1 | 4 | 33 |
|  | Tian 2024 | 42 | 1 | 24 | 72 |
|  | Wang 2022 | 17 | 4 | 14 | 6 |
|  | Xing 2020* | 12 | 6 | 32 | 163 |
|  | Yan 2020* | 38 | 0 | 0 | 13 |
|  | Yu 2021 | 10 | 0 | 13 | 14 |
|  | Yuan 2024 | 44 | 8 | 20 | 39 |
|  | Zhang 2020 | 61 | 1 | 27 | 70 |
|  | Zhang 2023 | 40 | 3 | 25 | 22 |
|  | Zhao 2020 | 49 | 21 | 34 | 0 |
|  | Zhu 2022 | 50 | 44 | 4 | 346 |
| CMTs | Benoit 2024 | 101 | 53 | 119 | 862 |
|  | Deng 2023 | 9 | 18 | 16 | 5 |
|  | Gan 2022 | 29 | 0 | 17 | 148 |
|  | Lin 2021 | 13 | 0 | 21 | 16 |
|  | Lin 2023 | 1 | 0 | 9 | 163 |
|  | Tian 2024 | 20 | 0 | 46 | 73 |
|  | Wang 2022 | 10 | 1 | 21 | 9 |
|  | Yu 2021 | 8 | 0 | 15 | 14 |
|  | Yuan 2024 | 17 | 2 | 47 | 45 |
|  | Zhang 2020 | 19 | 0 | 69 | 71 |
| mNGS: Metagenomic next-generation sequencing; CMTs=Conventional microbiological tests; TP: True positive; FP: False positive; FN: False negative; TN: True negative  *= data was extracted from definite cases of *Mycobacterium tuberculosis* (TB) | | | | | |

**Supplementary Appendix 4.** Raw data of included tuberculosis meningoencephalitis studies with clinical diagnosis

| Diagnostic  Test | First Author, Year | TP | FP | FN | TN |
| --- | --- | --- | --- | --- | --- |
| mNGS | Chen 2022* | 74 | 0 | 14 | 128 |
|  | Lin 2021 | 20 | 0 | 14 | 16 |
|  | Lin 2024* | 11 | 0 | 3 | 14 |
|  | Wang 2022 | 17 | 4 | 14 | 6 |
|  | Xing 2020* | 12 | 6 | 32 | 163 |
|  | Yan 2020* | 38 | 0 | 0 | 13 |
|  | Yu 2021 | 10 | 0 | 13 | 14 |
| CMTs** | Lin 2021 | 13 | 0 | 21 | 16 |
|  | Wang 2022 | 10 | 1 | 21 | 9 |
|  | Yu 2021 | 8 | 0 | 15 | 14 |
| mNGS: Metagenomic next-generation sequencing; CMTs=Conventional microbiological tests; TP: True positive; FP: False positive; FN: False negative; TN: True negative  *= data was extracted from definite cases of *Mycobacterium tuberculosis* (TB)  **= in studies that used multiple CMT methods, we used the method that had the highest sensitivity to compare the mNGS | | | | | |

**Supplementary Appendix 5.** Forest plot showing the sensitivity and specificity of metagenomic next-generation sequencing (mNGS) for detecting infectious meningoencephalitis in patients


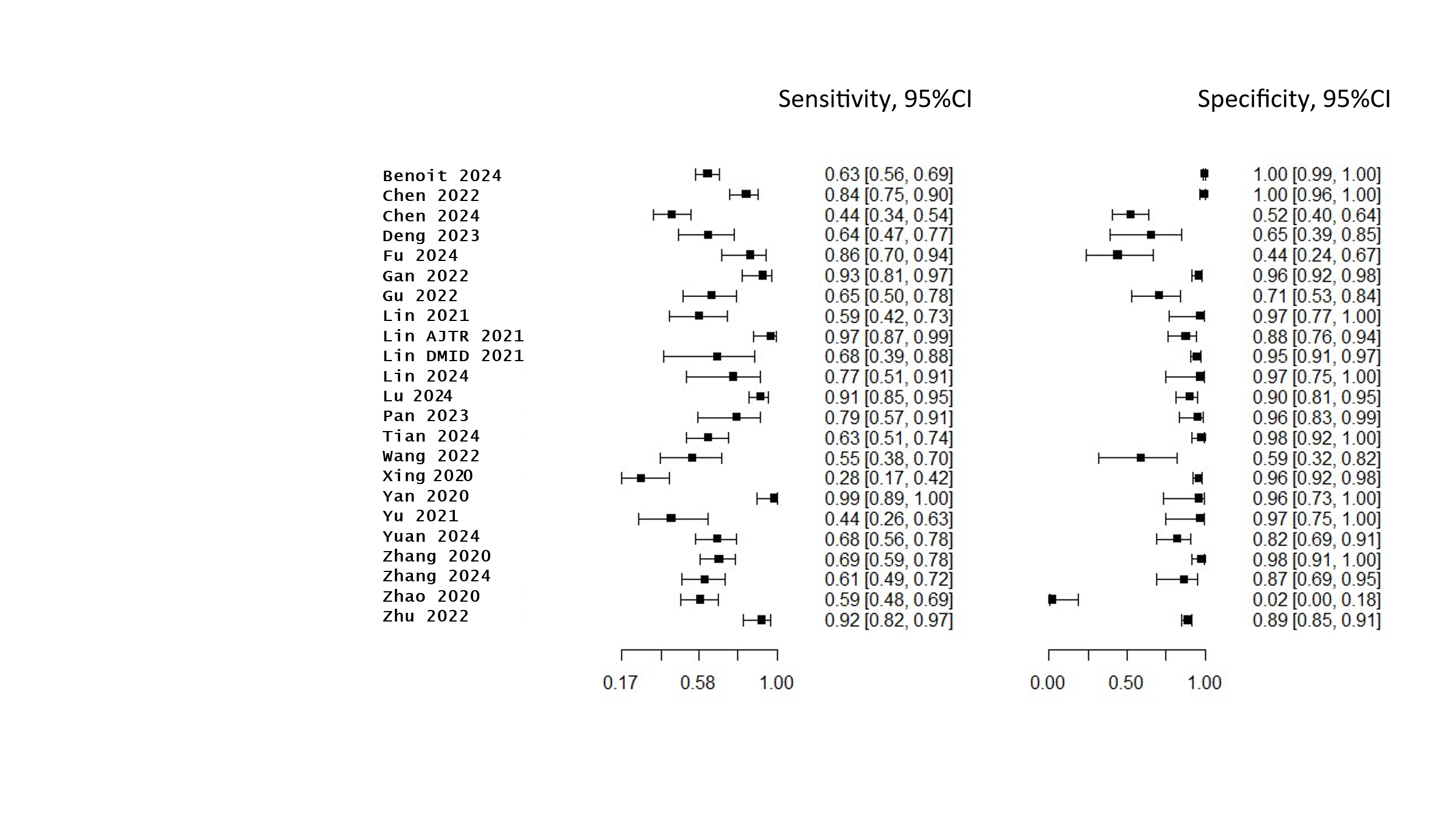


**Supplementary Appendix 6.** Forest plot showing the sensitivity and specificity of conventional microbiological tests (CMTs) for detecting infectious meningoencephalitis in patients


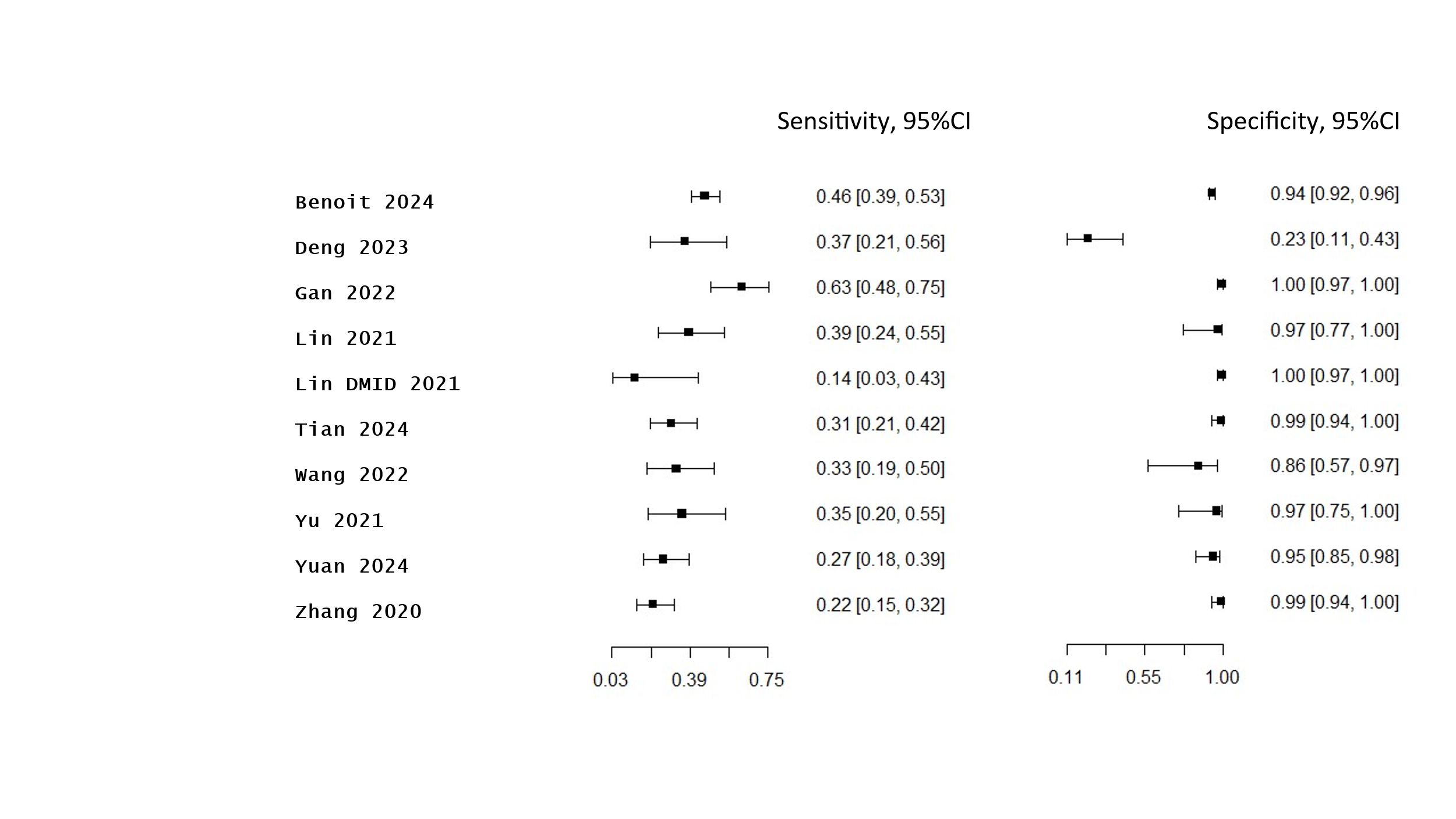

Supplement: ofaf274_Supplementary_Data [file ofaf274_supplementary_data.docx]
